# The Coronavirus Disease 2019 Spatial Care Path: Home, Community, and Emergency Diagnostic Portals

**DOI:** 10.3390/diagnostics12051216

**Published:** 2022-05-12

**Authors:** Gerald J. Kost

**Affiliations:** Fulbright Scholar 2020–2022, ASEAN Program, Point-of-Care Testing Center for Teaching and Research (POCT•CTR), Pathology and Laboratory Medicine, School of Medicine, University of California, Davis, CA 95616, USA; geraldkost@gmail.com; Tel.: +1-530-574-3945

**Keywords:** Emergency Use Authorization (EUA), endemic, false omission rate (R_FO_), home testing, point-of-care testing (POCT), positive predictive value geometric mean-squared (PV GM^2^), prevalence boundary, recursive protocol, tier, visual logistics

## Abstract

This research uses mathematically derived visual logistics to interpret COVID-19 molecular and rapid antigen test (RAgT) performance, determine prevalence boundaries where risk exceeds expectations, and evaluate benefits of recursive testing along home, community, and emergency spatial care paths. Mathematica and open access software helped graph relationships, compare performance patterns, and perform recursive computations. Tiered sensitivity/specificity comprise: (T1) 90%/95%; (T2) 95%/97.5%; and (T3) 100%/≥99%, respectively. In emergency medicine, median RAgT performance peaks at 13.2% prevalence, then falls below T1, generating risky prevalence boundaries. RAgTs in pediatric ERs/EDs parallel this pattern with asymptomatic worse than symptomatic performance. In communities, RAgTs display large uncertainty with median prevalence boundary of 14.8% for 1/20 missed diagnoses, and at prevalence > 33.3–36.9% risk 10% false omissions for symptomatic subjects. Recursive testing improves home RAgT performance. Home molecular tests elevate performance above T1 but lack adequate validation. Widespread RAgT availability encourages self-testing. Asymptomatic RAgT and PCR-based saliva testing present the highest chance of missed diagnoses. Home testing twice, once just before mingling, and molecular-based self-testing, help avoid false omissions. Community and ER/ED RAgTs can identify contagiousness in low prevalence. Real-world trials of performance, cost-effectiveness, and public health impact could identify home molecular diagnostics as an optimal diagnostic portal.

## 1. Introduction

Now in the third year of the Coronavirus disease 19 (COVID-19) pandemic, we observe worldwide proliferation of novel COVID-19 diagnostic tests. Proliferation of COVID-19 commercial diagnostics authorized with limited clinical validation; emergence of highly contagious Omicron, BA.2, and other variants and sub-variants; distribution of one billion rapid antigen tests (RAgTs) [1,2]; the new White House “test (in pharmacies) and treat” program [3]; and relaxed preventative measures, such as safe spacing and masking, call for analysis of COVID-19 test performance along spatial care paths where people choose their own testing options—that is, select their own “diagnostic portals.” A spatial care path is the most efficient route taken by individuals and patients when receiving care in the healthcare small-world network of home, community, and emergency medicine settings [4,5,6].

Prevalence, the percentage of a population affected with COVID-19 at a given time, can vary unpredictably in different locales, because of severe acute respiratory syndrome Coronavirus 2 (SARS-CoV-2) variants, super spreaders, asymptomatic carriers, migrating hotspots, episodic re-openings, incomplete testing, delayed reporting, and other factors, such as erratic sampling and marginal reliability of rushed-to-market COVID-19 tests. Clinical performance depends on whether there are symptoms or not, the patient’s viral load, and the timing, location, and quality of the environment, specimen collection (e.g., saliva, and anterior nasal, mid-turbinate, or nasopharyngeal swab), and testing method.

Nonetheless, a large surge in clinical evaluations published recently paints a clear picture of what to expect. Expectations are at the heart of public acceptance and empowerment. Americans can choose from an array of self-testing options, use COVID-19 tests mailed to them at no cost by the government, and engage several different diagnostic portals. Future public health practices will hinge on fundamental understanding of how point-of-need testing meets or defeats attempts to keep families safe, temper contagiousness of new variants, safeguard schools and workplaces, and transition smoothly forward.

Therefore, the main goal of this article is to facilitate informed selection of diagnostic tests when faced with the multi-dimensional challenges of fluctuating endemic disease, newly emerging variants, increasing prevalence, and variably accessible testing options with complex performance patterns. Another goal is to minimize false omissions, that is, missed diagnoses that unknowingly elevate risk, create local recurrences, spread contagion, and adversely interrupt personal life, community activities, and work productivity.

## 2. Methods and Materials

### 2.1. Emergency Use Authorizations

Assays should be well balanced; that is, they should achieve both high sensitivity and high specificity. Adverse patterns identify tests that do not perform well but have received Food and Drug Administration (FDA) Emergency Use Authorization (EUA). Positive percent agreement (PPA) and negative percent agreement (NPA) data were extracted from FDA lists of EUAs [7] for home RAgTs and home LAMP (loop-mediated isothermal amplification) tests collated up to the beginning of January 2022. In the Supplementary Materials at the end of this article, Table S1 lists EUA PPA and NPA claims. Donato et al. [8] provided the only independent clinical evaluation of sensitivity and specificity for a LAMP molecular home self-test found during searches.

### 2.2. Clinical Evaluations

A total of 82 clinical studies were tabulated. Table 1 summarizes performance metrics for the primary study groups (left column) and lists supporting tabulations (right column) found in the Supplementary Materials. Papers generated by PubMed, other searches, and bibliographies of systematic reviews and meta-analyses comprised (a) 34 clinical evaluations [9,10,11,12,13,14,15,16,17,18,19,20,21,22,23,24,25,26,27,28,29,30,31,32,33,34,35,36,37,38,39,40,41,42] of the use of RAgTs in communities (see Table S2); (b) 30 clinical evaluations [43,44,45,46,47,48,49,50,51,52,53,54,55,56,57,58,59,60,61,62,63,64,65,66,67,68,69,70,71,72] of RAgTs applied in emergency medicine (emergency rooms and emergency departments), including 9 with results for pediatric patients [58,65,66,67,68,69,70,71,72] (Table S3); and (c) 18 clinical evaluations [73,74,75,76,77,78,79,80,81,82,83,84,85,86,87,88,89,90] that reported results for PCR-based testing of saliva in community groups of strictly asymptomatic subjects (Table S4). No human subjects were involved in this research.

### 2.3. Sensitivity and Specificity Metrics

Sensitivity and specificity metrics from community (Table S2) and emergency medicine evaluations (Table S3) were subdivided into symptomatic and asymptomatic groups (see Table 1). Merging raw data was not practical in light of heterogeneity, missing elements, unbalanced study designs, and inconsistent reporting in the clinical evaluations. Some studies were reported prior to peer review by medRxiv and preliminarily in various journals. In essence, each study generated results reflected in sensitivity and specificity median performance for each clinical setting.

### 2.4. Bayesian Mathematics and Performance Tiers

Please refer to open access papers by Kost [91,92,93] in the *Archives of Pathology & Laboratory Medicine* for descriptions of mathematical methods, visual logistics, computational design, and open access software. Table S5 lists Bayesian equations derived to generate the graphics displayed in this paper. Table 2 presents the mathematical design criteria for the three tiers, which are intended to systematically harmonize Bayesian post hoc performance criteria. The design criteria encompass and simultaneously integrate the performance level, sensitivity, specificity, target prevalence boundary, and false omission rate, R_FO_, which reflects risk of missed diagnoses.

### 2.5. Prevalence Boundaries

A prevalence boundary is defined as the prevalence at which the rate of false omissions, R_FO_, exceeds a specified risk tolerance, such as 5% (1 in 20 diagnoses missed) or 10% (1 in 10 missed). Please note that R_FO_ = 1 − (Negative Predictive Value) [Equation (20)] in Supplementary Materials. Prevalence boundary is calculated using Equation (26) in Table S5 and is apparent where the R_FO_ curve intersects the horizontal line demarcating risk tolerance. Users can select the level of risk based on COVID-19 management and mitigation objectives.

The sensitivity needed to achieve a desired prevalence boundary given the specificity, R_FO_, and prevailing prevalence can be calculated using Equation (27a,b) in Supplementary Materials (newly derived). For example, if the prevalence is 50.6%, and you do not want to miss more than 1 in 20 diagnoses of COVID-19 (R_FO_ = 5%), then given a specificity of 97.5%, Equation (26a) in Supplementary Materials predicts you will need a test with at least 95% sensitivity, that is, Tier 2 performance (see Table 2).

### 2.6. Pattern Recognition

Visual logistics reveal performance patterns and diagnostic pitfalls over the entire range of prevalence. The approach to pattern recognition presented here, called “predictive value geometric mean-squared (PV GM^2^),” is strictly visual [91,92,93]. The PV GM^2^ curve represents a distinguishing “fingerprint” of performance, and thus far in this research, no two fingerprints have coincided. PV GM^2^ curves visualize how low (≤20%), moderate (20–70%), and high (≥70%) prevalence affect diagnostic performance in a single continuous graphic.

Point values of PV GM^2^ at fixed prevalence should not be compared because of potential duplicity of the values at different levels of prevalence. Unrealistic comparisons (e.g., test sensitivity 10% and specificity 100%) should be avoided as they produce meaningless curves. The point of PV GM^2^ visualization is to differentiate performance patterns for tests achieving at least Tier 1 sensitivity and specificity criteria (see Table 2), or if below Tier 1 (“subtier”), then to understand why and where performance fails and crosses below the Tier 1 threshold. 

### 2.7. Recursion

The recursive formulas for positive predictive value (PPV) [Equation (22a)] in Supplementary Materials and negative predictive value (NPV) [Equation (22b)] in Supplementary Materials allow calculations of predictive value geometric mean-squared (PV GM^2^) and R_FO_ performance for repeat testing. When testing only twice with the same assay, single equations can be derived to graph recursive PV GM^2^ and R_FO_ versus prevalence from 0 to 100% and to conveniently determine graphically the recursive prevalence boundaries at user-defined risk levels of 5% and 10% for missed diagnoses.

## 3. Results

**Figure 1:**
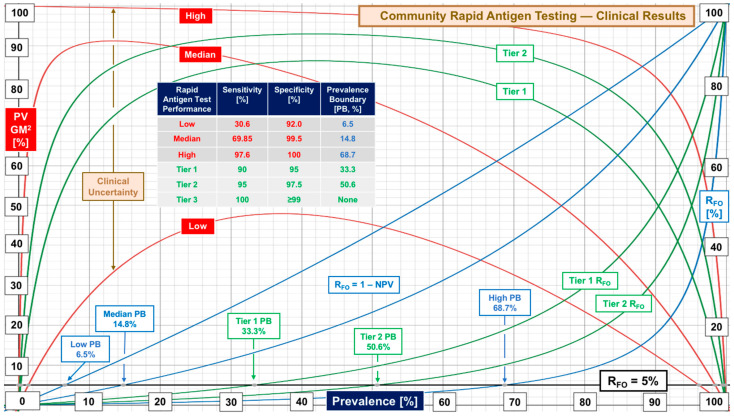
Performance of rapid antigen testing in community settings. Predictive value geometric mean-squared (PV GM^2^) curves reflect performance (left vertical axis) over the entire range of prevalence (horizontal axis). R_FO_, the false omission rate (right axis), indicates the frequency of missed diagnoses. PV GM^2^ and R_FO_ curves are plotted for low, median, and high sensitivity/specificity documented in published clinical studies conducted in community settings (see Supplementary Materials). Performance Tiers 1 and 2 are plotted in green. The black horizontal line represents a risk level of 5% where 1 in 20 diagnoses will be missed. A prevalence boundary (PB) is the point at which the risk of a missed diagnosis exceeds the threshold of 1 in 20, that is, the point at which risk of contagion rises significantly and quickly.

**Figure 2:**
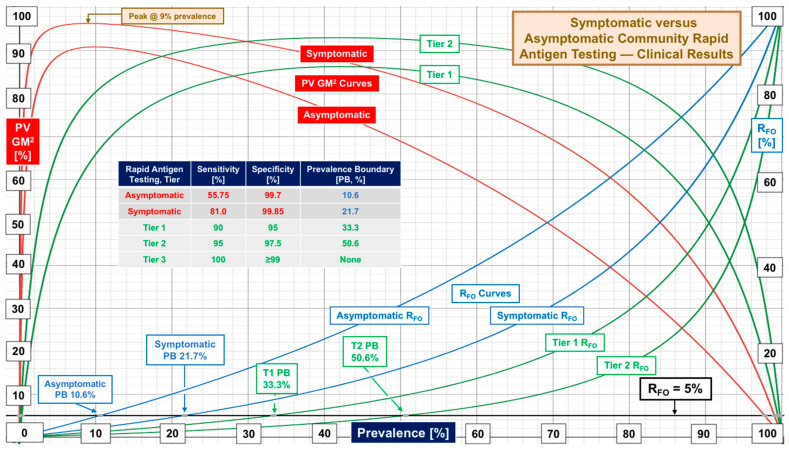
Comparison of symptomatic versus asymptomatic community rapid antigen testing performance based on evidence.

Figure 1 illustrates patterns of low, high, and median performance documented by evaluations of RAgTs conducted in communities of several countries and states in the United States. RAgTs display large uncertainty with a median prevalence boundary of 14.8% for 1 in 20 missed diagnoses (R_FO_ 5%). Median sensitivity of 69.85% (range 30.6–97.6%) explains the rapid fall off of PV GM^2^ due to increasing false negatives as prevalence increases, while median specificity achieves Tier 3. Figure 2 compares performance for asymptomatic and symptomatic subjects, the latter showing peak performance at 9% prevalence and a prevalence boundary of 21.7% (at 5% R_FO_). Median sensitivity for symptomatic subjects was 81.0%. For automated instrument antigen tests, it is 73% (see Table 1 and Table S2).

**Figure 3:**
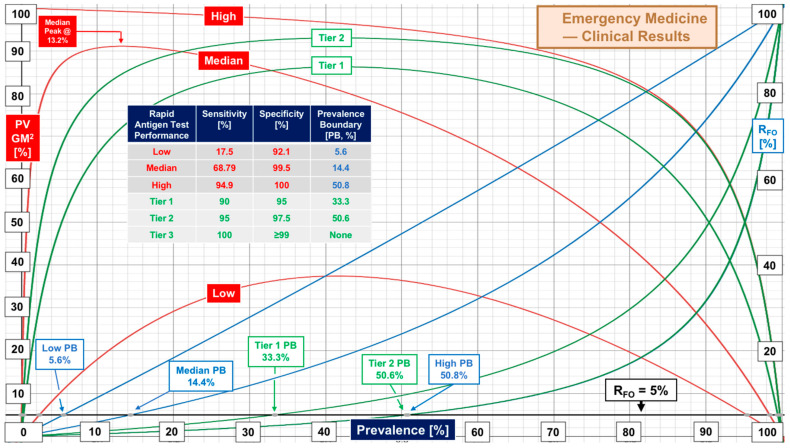
Real-world performance of rapid antigen testing in emergency medicine.

**Figure 4:**
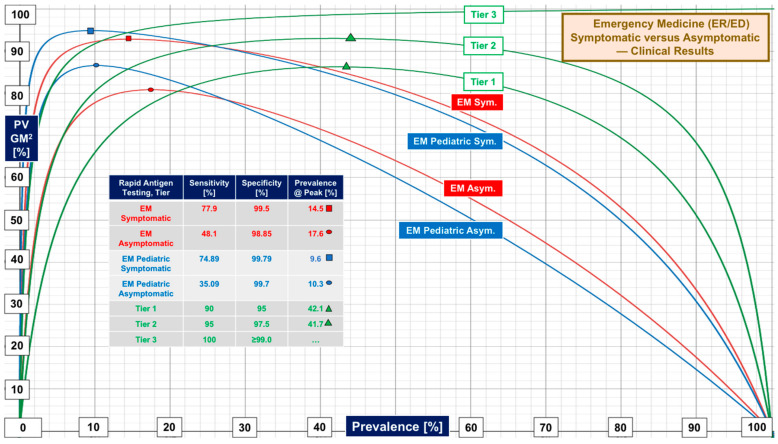
Comparison of symptomatic versus asymptomatic rapid antigen test performance in emergency medicine. The blue PV GM^2^ curves illustrate RAgT performance for pediatric patients presenting to emergency rooms (ERs) and emergency departments (EDs). The curves show that RAgTs are “unbalanced” in contrast to the design of the performance tiers. Please see the legend in the inset table for prevalence at points of best performance. For asymptomatic patients, performance falls off quickly as prevalence increases, which limits use in emergency medicine settings where, through self-selection by sick patients, one would expect prevalence to be higher than in the community. COVID-19 detection is more likely in symptomatic patients, in part because of higher viral loads.

Figure 3 presents patterns of RAgT performance for evaluations conducted in emergency medicine settings [emergency rooms (ERs) and emergency departments (EDs)] (see Table 1 and Table S3). Median sensitivity of 68.79% and specificity of 99.5% generate peak performance at 13.2% prevalence. The prevalence boundary for R_FO_ of 5% was 14.4%, almost identical to that seen in Figure 1. Figure 4 compares performance for symptomatic and asymptomatic general populations and children seen in ERs and EDs, with the former marginally better than the latter. The right column of the inset table lists prevalence at peak performance.

**Figure 5:**
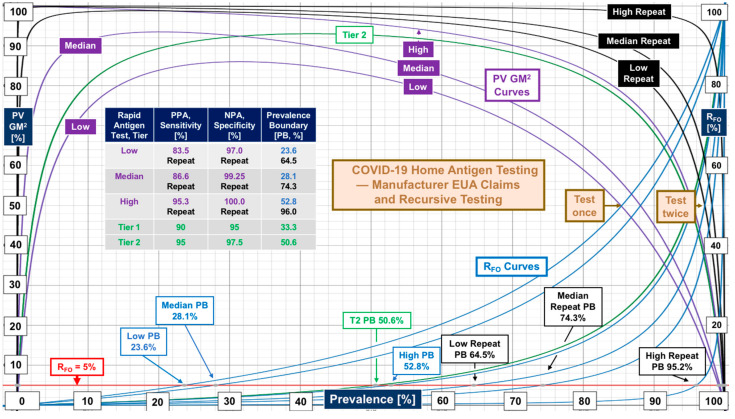
Performance of rapid antigen testing for home self-testing based on manufacturer claims in FDA EUA authorizations. Theoretical analysis of manufacturer claims shows that repeat testing yields higher performance and prevalence boundaries. Median recursive performance achieves Tier 2. The median recursive prevalence boundary of 74.3% reflects a reasonable minimum for Omicron, BA.2, and other emerging variants and sub-variants.

**Figure 6:**
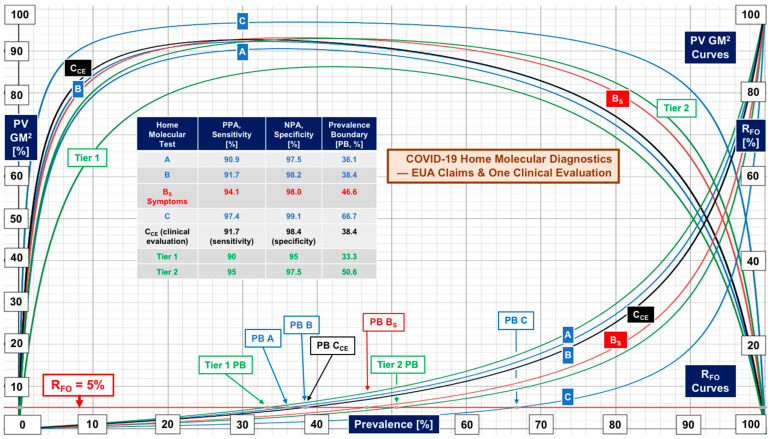
Performance of molecular diagnostics for home self-testing. In the case of home molecular diagnostics, one independent clinical evaluation, shown by the “**C_CE_**” curves, achieved Tier 1 performance.

There were no evaluations conducted directly in homes for EUA tests with real-world data generated by laypersons who perform the self-testing. Therefore, Figure 5 and Figure 6 illustrate performance based on manufacturer claims in information for users (IFUs) documents. Figure 5 also illustrates the theoretical improvement in performance achievable by repeat testing, typically within three days, as described in IFUs. Manufacturers made no claims regarding recursive testing. As can be seen in Figure 5, repeat testing pushes performance up to Tier 2. Figure 6 shows individual performance for three home molecular (LAMP) self-tests, as claimed by manufacturers. One independently conducted clinical evaluation by Donato et al. [8] was found. Real-world evidence revealed Tier 1 performance (curve “**CCE**”) versus the claim of Tier 2.

**Figure 7:**
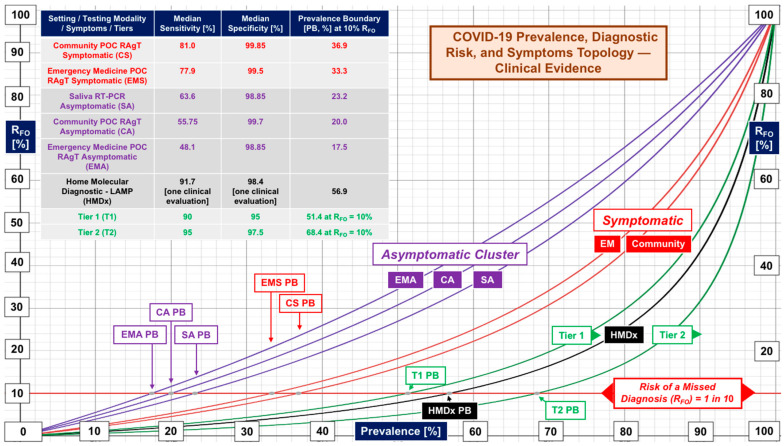
False omission rate topology for asymptomatic and symptomatic subjects. The purple cluster shows performance for asymptomatic subjects in emergency medicine (EMA) and community (CA) settings, while the red cluster reflects results for symptomatic subjects. Saliva testing for asymptomatic subjects (SA, purple), with specimen collection at points of need and PCR analysis performed in laboratories, did not differ substantially from rapid antigen testing. “**HMDx**” represents one clinical evaluation of molecular self-testing, which achieved performance between Tier 1 and Tier 2. In this case the risk of a missed diagnosis (R_FO_) is 10%, shown by the red horizontal line.

Figure 7 summarizes the foregoing results from a risk perspective by plotting R_FO_ versus prevalence with the threshold of a missed diagnosis at 1 in 10 (R_FO_ 10%). Recall, R_FO_ = 1 − NPV. Interestingly, the curves group into clusters for asymptomatic (purple) and symptomatic (red) subjects, while the Donato et al. [8] evaluation of home molecular testing (curve “**HMDx**”) demonstrated a Tier 1 prevalence boundary of 56.9%. Even at a relatively high 10% risk of missed diagnoses, prevalence boundaries for asymptomatic subjects are low (17.5–23.2%), including for self-collected saliva specimens obtained in community sites from asymptomatic subjects with PCR-based testing performed later, typically within 24–72 h in reference laboratories (see Table 1 and Table S4).

## 4. Discussion

### 4.1. Missed Diagnoses

SARS-CoV-2 prevalence in South African blood donors skyrocketed to 71% even before the Omicron-driven wave arrived [94]. This variant peaked in the United States, only to be followed by the more contagious BA.2 variant. Omicron is sweeping Southeast Asia. For example, Thailand has reported over 50,000 new cases per day, and Vietnam, 120,000. These outbreaks bump prevalence to levels requiring Tier 2 performance to avoid excessive false negatives.

Mathematical transformation of pre-test to post-test probability of COVID-19 [92,93] allows computation of R_FO_, the false omission rate [Equation (20), Table S5], and determination of the prevalence boundary (PB) [Equation (26)] in Supplementary Materials. Shallow PBs limit the clinical usefulness of RAgTs, because of excessive missed diagnoses. If one knows test specificity, sets the R_FO_ threshold (e.g., 5 or 10%), and establishes the PB appropriate for local prevalence, then Equation (27a) in Supplementary Materials can be used to calculate the minimum test sensitivity required. Frequent false omissions result in stealth spread of disease.

### 4.2. Transparency

Unfortunately, there is no way of singling out infectious patients who have false negative test results without repeat testing or additional evaluation. Using quantitative high sensitivity synchrotron X-ray fluorescence imaging, Koller et al. [95] showed that qualitative visual read-outs miss immobilized antigen-antibody-labeled conjugate complexes of SARS-CoV-2 signals on lateral flow detection devices. On the other hand, one report showed that ~92% of patients infected with SARS-CoV-2, but missed by antigen testing, contained no viable virus [96]. Even the performance of a so-called “ultra-sensitive” antigen test, after exclusion of samples with PCR cycle threshold >35, hovered around Tier 2 for PPA and below it for NPA [97].

There is no explanation for why the PPA and NPA of assays documented in FDA EUAs have not progressively improved. However, the FDA has not required improvement. Liberal FDA approval seems to have diminished competition. One wonders what will become of subtier rapid antigen tests in a competitive market following the end of the “EUA era.” People purchasing test kits or ordering them free from COVIDtests.gov should receive disclosure of specificity (to rule-in COVID-19) and sensitivity (to rule it out) documented in clinical evaluations, and may need interactive apps to access prevailing prevalence.

The “…successful implementation of rapid antigen testing protocols must closely consider technical, pre-analytical, analytical and clinical assay performance and interpret and verify test results depending on the pretest-probability of SARS-CoV-2 infection” [98]. With that advice, the International Federation of Clinical Chemistry (IFCC) COVID-19 Task Force underscored the need for careful analysis of how prevalence impacts antigen test results in different clinical settings. *Consumer Reports* and other public advocacy groups may eventually compare and rank commercially available point-of-care antigen and saliva tests for the public.

### 4.3. Public Health at Points of Need

The death toll of the pandemic, including excess deaths from neglect, is estimated to be as high as 18.2 million [99] or even higher. The COVID-19 crisis has confirmed and expanded worldwide what we learned during Ebola virus disease outbreaks in West Africa. There is unequivocal need for point-of-care testing [4,5,6,100,101,102,103,104,105]. People in the United States now have access to COVID-19 RAgTs and molecular diagnostics online, by mail, and in neighborhood stores. With ubiquitous access comes responsibility—on the part of academics, public health educational institutions, professional societies, governments, industry, and global organizations to promote high quality testing. 

The CORONADx Project in the European Union [106] is planned to produce affordable “PATHAG,” ultra-rapid COVID-19 antigen test strips for first-line screening; “PATHPOD,” portable LAMP detection for mobile clinics and community health centers; and “PATHLOCK,” kit detection using CRISPR-Cas13 technology. This initiative will add to the massive expansion of point-of-care strategies and promote higher quality molecular self-testing [1,2,104,105]. Ready access to rapid testing along spatial care paths from home to hospital raises public expectations for controlling transmission, combatting COVID-19, and forestalling future pandemics.

Children, teens, and young adults, even if vaccinated, may quietly spread disease, especially now that the more infectious variants and sub-variants are elevating community prevalence. Complicating matters, hospitals in some limited resource countries administer fake vaccinations (injecting water) for monitory gain [107]. Nonetheless, the CDC views vaccination rates as indicators of pandemic status worldwide [108], a reasonable position confirmed by Omicron surges in Hong Kong and China where vaccination rates have been low and vaccines less effective.

Vaccination attenuates the severity of disease but does not necessarily eliminate SARS-CoV-2 infection. Thus, individuals and their medical providers face the daunting challenge of guessing local and regional prevalence in order to interpret test results. Public health officials can help by periodically documenting prevalence and by encouraging self-testing in communities and directly within homes. Pattern recognition by means of PV GM^2^ and R_FO_ curves allows healthcare providers to quickly tailor the quality of testing to needs.

### 4.4. Focus, Standardization, and Risk Management

RAgTs now are ubiquitous worldwide. They enable people to test frequently and inexpensively wherever they wish. Progressive societal “normalization” increases demand for convenient, fast, and inexpensive test results for decision making in various settings comprising public gatherings, communities, homes, schools, workplaces, factories, convalescent care, prisons, university campuses, sports events, travel, airports, rural regions, and limited-resource settings abroad.

Figure 2, Figure 4 and Figure 7 show RAgTs detect SARS-CoV-2 infections in symptomatic subjects more effectively than asymptomatic, for whom community screening using PCR-based saliva testing offers no significant diagnostic advantage (see Figure 7). Asymptomatic RAgT and PCR-based saliva testing present the highest risk of missing diagnoses when highly contagious new variants increase prevalence. Figure 7 provides an essential “bottom line” for risk assessment. It illustrates the advantages of using the false omission rate (R_FO_) and prevalence boundary (PB) as criteria for the selection an appropriate diagnostic portal in the context of local prevalence. 

Repeat rapid antigen home testing with the second test just before mingling is theoretically sound (see Figure 5) and is encouraged in commercial products containing two tests for screening but has not been validated. Home molecular testing one time shows promise (see “**HMDx”** in Figure 7). Controlled studies using some of the nationally distributed free tests could determine the efficacy of home self-testing, and should encompass both RAgT and LAMP assays, as well as investigation of the effectiveness of repeat testing and whether the second test should be independent (orthogonal) [93].

Weaknesses in COVID-19 rapid antigen test performance, even for products introduced more than one year after the FDA first started granting COVID-19 EUAs, call for standardization, or at least a process for attaining consistency and improving sensitivity. The contrast in performance based on FDA EUA PPA and NPA metrics versus real-world sensitivity and specificity from clinical investigations (Figure 1, Figure 2, Figure 3 and Figure 4) demands multicenter studies with diverse populations, well defined clinical settings, different age groups, and large sample sizes.

## 5. Conclusions

Widespread availability of RAgTs encourages self-testing, but people receiving millions of free RAgTs for self-testing need guidance. If public health educational institutions and practitioners adapt, learn, teach, and incorporate proven point-of-care strategies, they will better mitigate variant surges and the future outbreaks [105,109,110,111]. RAgTs facilitate transitioning risk avoidance to risk management, especially in limited-resource settings where people cannot afford extended lockdowns, expensive PCR tests with delayed results, and loss of employment. For those, we recommend mobile testing in vans and inexpensive sample collection kiosks at sites of need [112].

Subtier tests should be improved or retired from FDA EUA status, because of poor performance, uncertainty, and false omissions that increase exponentially with increasing prevalence. Every step beyond a prevalence boundary magnifies chances of missing a diagnosis (see Figure 7). Tier 3, with its 100% sensitivity, could eliminate false omissions and prevalence boundaries. However, Tier 3 appears out of reach for the current generations of RAgTs. The FDA should tighten authorization criteria and shift the evaluation paradigm from template-based to clinical proof.

Tier 2 performance represents an attainable, practical, and sustainable standard of excellence, in view of the fact that several EUA manufacturers have claimed that performance threshold (see Supplemental Figure S1). The scheme in Table 1 can be used to establish constraints on 95% confidence limits and reduce uncertainty. Supportive actions the FDA should take comprise (a) tightening authorization thresholds and integrating prevalence boundaries, (b) increasing sample sizes to generate robust confidence intervals, (c) requiring comparison of symptomatic versus asymptomatic patients, (d) validating environmental limits of reagents, (e) publishing post-market follow-up of performance. 

Testing in homes and communities makes sense. When acute symptoms are present, the analyses in this paper shows that rapid response use of RAgTs in communities and ERs/EDs can mitigate spread by helping to immediately identify acute contagiousness in low to moderate prevalence. Real-world trials of repeat testing, diagnostic cost-effectiveness, and public health impact could identify home molecular diagnostics as an optimal diagnostic portal for self-testing and rapid decision making at most levels of prevalence. Point-of-care testing has and will continue to provide a valuable resource for crisis response in the current pandemic and whatever the future brings.

## Figures and Tables

**Table 1:** Performance Metrics for Home, Community, and Emergency Medicine COVID-19 Testing.

Clinical Space	Median [N, Range]	Median [N, Range]	Data Source
**Home Self-Tests**			**Supplemental Table S1**
Rapid antigen tests	PPA 86.6 [12, 83.5–95.3]	NPA 99.25 [12, 97–100]	Manufacturer FDA EUA claim (not substantiated)
Isothermal (LAMP) molecular tests	PPA 91.7 [3, 90.9–97.4]	NPA 98.2 [3, 97.5–99.1]	Manufacturer FDA EUA claim (not substantiated)
Isothermal (LAMP) molecular test	Sensitivity 91.7 [1, CI NR]	Specificity 98.4 [1, CI NR]	One independent clinical evaluation, see Donato et al. [8]
**Community RAgTs**	**Sensitivity**	**Specificity**	**Supplemental Table S2**
Overall	69.85 [24, 30.6–97.6]	99.5 [24, 92–100]	Performance evaluations
Symptomatic	81.0 [19, 47.7–96.5]	99.85 [16, 85–100]	Symptomatic subjects
Asymptomatic	55.75 [20, 37–88]	99.70 [16, 97.8–100]	Asymptomatic subjects
Automated antigen tests-overall	62.3 [3, 43.3–100]	99.5 [3, 94.8–99.9]	Evaluations using automated laboratory instruments (small set)
-symptomatic	73 [3, 68.5–88.9]	100 [3, 100]	Symptomatic subjects for above
**Emergency Medicine RAgTs**	**Sensitivity**	**Specificity**	**Supplemental Table S3**
EM Overall	68.79 [20, 17.5–94.9]	99.5 [20, 92.1–100]	ER and ED evaluations
Symptomatic	77.9 [15, 43.3–95.8]	99.5 [14, 88.2–100]	Symptomatic EM subjects
Asymptomatic	48.1 [11, 28.6–92.1]	98.85 [10, 92.3–100]	Asymptomatic EM subjects
Pediatric EM	71.3 [10, 42.9–94.1]	99.55 [10, 91.9–100]	Pediatric ER/ED patients only
Ped. Symptomatic	74.89 [6, 45.4–87.9]	99.79 [6, 98.5–100]	Symptomatic EM children
Ped. Asymptomatic	35.09 [2, 27.27–42.9]	99.7 [2, 99.4–100]	Asymptomatic EM children
**Saliva Testing**	**Sensitivity**	**Specificity**	**Supplemental Table S4**
Asymptomatic, molecular diagnostics	63.6 [19, 16.8–95]	98.85 [14, 95–100]	Community evaluations with strictly asymptomatic subjects

**Abbreviations:** CI, 95% confidence interval; ED, emergency department; EM, emergency medicine; ER, emergency room; EUA, Emergency Use Authorization; FDA, Food and Drug Administration; LAMP, reverse transcription loop-mediated isothermal amplification; NR, not reported; PPA, positive percent agreement; NPA, negative percent agreement; Ped., pediatric; POC, point of care; and RAgTs, rapid antigen tests.

**Table 2:** Performance Tiers with Coordinated and Integrated False Omission Rates and Prevalence Boundaries Bracketing Community Immunity from 50% to 85%.

Tier	Performance Level	Sensitivity, %	Specificity, %	Target Prevalence Boundary [Actual] at R_FO_ of 5% 10% 20%		
1	Low	90	95	33% (33.3)	50% (51.4)	70% (70.3)
2	Marginal	95	97.5	50% (50.6)	70% (68.4)	85% (83.0)
3	High	100	≥99	No Boundary	No Boundary	No Boundary

**Abbreviation:** R_FO_, false omission rate.

## Data Availability

All raw data are included in the paper and its Supplementary Materials.

## Supplementary Materials

The following supporting information can be downloaded at: https://www.mdpi.com/article/10.3390/diagnostics12051216/s1, Figure S1: Rate of False Omissions for FDA EUA COVID-19 Rapid Antigen Tests; Table S1: COVID-19 Tests with FDA Emergency Use Authorization for Home Self-testing; Table S2: COVID-19 Antigen Test Performance for Symptomatic and Asymptomatic Subjects in Community Settings; Table S3: COVID-19 Antigen Test Performance in Emergency Medicine; Table S4: COVID-19 Saliva Testing Sensitivity and Specificity Performance in Strictly Asymptomatic Sub-jects (no mixed populations)—Clinical Evidence; Table S5: Fundamental Definitions, Derived Equations, Ratios/Rates, Recursive Formulas, Predictive Value Geometric Mean-squared, and Prevalence Boundary; Table S6: Antigen Tests with FDA Emergency Use Authorization Ranked by Positive Percent Agreement.
