# Supplementary material for: The Coronavirus Disease 2019 Spatial Care Path: Home, Community, and Emergency Diagnostic Portals
Source: Diagnostics (Basel). 2022 May 12;12(5):1216. doi: 10.3390/diagnostics12051216 (PMC9140623; doi:10.3390/diagnostics12051216)
Supplement: Supplementary file 1 [file diagnostics-12-01216-s001.zip › diagnostics-1723038-supplementary.pdf]

diagnostics

MDPI

Table S1. COVID-19 Tests with FDA Emergency Use Authorization for Home Self-testing.

Category of Test, Notables	Tier (without Repetition), Sample Size	Company, EUA Latest Date LOA [Earliest Date LOA], Product Name	Company PPA (%) Claim [CI, Δ]	Company NPA (%) Claim [CI, Δ]	Assay Method, Specimen Type, Age, Time Interval Protocol for Specimen Collection, and Notes
**Part I. Antigen Tests**	**Sub-Tier** (8)				
Lowest PPA of 83.5%	N = 350	Quidel Corp. 10/21/21 [3/31/21] Quickview At-Home OTC COVID-19 Test	**83.5** [74.9–89.6, Δ = 14.7]	**99.2** [97.2–99.8, Δ = 2.6]	Lateral flow, visual read, NCP Ag. AN. ≥14 years or adult-collected ≥2 years. OTC. Home testing, serial screening ≥24 & ≤36 h between tests.
Smallest Δ uncertainty of 13.8% for the PPA 95% CI	N = 460	Abbott Diagnostics 1/7/22 [3/31/21] BinaxNow COVID-19 Antigen SelfTEST (OTC)	**84.6** [76.8–90.6, Δ =13.8]	**98.5** [96.6–99.5, Δ = 2.9]	SelfTEST: Lateral flow, visual read, NCP Ag. AN. ≥15 years or adult-collected ≥2 years. Self-swab and self-test 2 times ≥24, ≤48 h apart. Home testing. PPA & NPA established from single test ≤7 days from symptom onset.
Largest sample size, smallest Δ uncertainty of 1% for the NPA 95% CI	N = 597	BD (Becton Dickinson) 11/23/21 [8/24/21] Veritor At-Home COVID-19 Test	**84.6** [70.3–92.8, Δ = 22.5]	**99.8** [99–100, Δ = 1]	Chromatographic, digital immunoassay, NCP Ag. Smartphone read using Scanwell Health App. Anterior nares. ≥14 years or adult-collected ≥2 years within 7 days. Home testing. With or without symptoms when test twice ≥24 & ≤48 h between tests. OTC.
	N = 165 (19 children added 9/21)	Orasure 1/27/22 [6/4/21] InteliSwab COVID-19 Rapid Test (OTC)	**85** [74–92, Δ = 21]	**98** [93–100, Δ = 6]	Lateral flow, visual read, NCP Ag. AN Ag. ≥18 years or children≥2 years if adult-collected. OTC. Home testing, serial 2-test screening ≥24 & ≤36 h between tests.
NPA 100%, FP 0, and PPV 100%.	N = 257	InBios 1/25/22 [11/22/21] SCoV-2 Ag Detect Rapid Self-Test	**85.71** [70.62–93.74, Δ = 23.12]	**100** [98.30–100.00, Δ = 1.70]	Lateral flow, visual read, NCP Ag. AN. Home testing, serial screening ≥14 years or adult-collected ≥2 years within 5 days of symptoms. With or without symptoms when test twice ≥24 & ≤48 h between tests. OTC.
	N = 268	Siemens 2/9/22 [12/29/21] CLINITEST Rapid COVID-19 Ag Self-Test	**86.5** [79.6–91.3, Δ = 11.7]	**99.3** [95.9–100, Δ = 4.1]	Lateral flow, visual read. NCP Ag. Anterior nares. OTC. Home testing, serial screening ≥14 years or adult-collected ≥2 years within 7 days of symptoms. With or without symptoms when test twice ≥24 & ≤48 h between tests.

Lot COVGCCM0008 was recalled due to FPs 12/28/21.	N = 492	Celltrion DiaTrust COVID-19 Ag Home Test 10/21/21 [10/21/21]	**86.7** [73.8–93.7, Δ = 19.9]	**99.8** [98.7–100.0, Δ = 1.3]	Lateral flow, visual read. OTC. Mid-turbinate swab. Home testing, serial screening "twice over two or three days" with ≥24 & ≤48 h between tests with or without symptoms. Subjects ≥14 years old.
Largest Δ uncertainty of 25% for the PPA 95% CI	N = 153	AccessBio 1/22/22 [8/2/21] CareStart COVID-19 Antigen Home Test	**87** [70–95, Δ = 25]	**98** [93–99, Δ = 6]	Lateral flow, visual read, NCP Ag. OTC. AN. ≥14 years or adult-collected, ≥2 years. Home testing, serial screening, With or without symptoms when test twice ≥24 & ≤48 h between tests.
	**Tier 1** (3)				
NPA 100%, FP 0, and PPV 100%.	N = 172	Acon Laboratories 10/19/21 [10/4/21] Flowflex COVID-19 Antigen Home Test	**93** [81–99, Δ = 18]	**100** [97–100, Δ = 3]	Lateral flow, visual read, NCP Ag. OTC. AN. ≥14 years or adult-collected, ≥2 years. Performed once <7 days of symptom onset or without symptoms or other epidemiological reasons.
Largest Δ uncertainty of 6.2% for the NPA 95% CI	N = 139	iHealth Labs 12/22/21 [11/5/21] COVID-19 Antigen Rapid Test	**94.3** [81.4–98.4, Δ = 17.0]	**98.1** [93.3–99.5, Δ = 6.2]	Lateral flow, visual read, home testing, serial screening. OTC. AN. <7 days of symptoms onset for 15 years, adult-collected ≥2 years. Without symptoms performed twice >24, <48 h between tests.
First FDA EUA. High PPA and lowest NPA, i.e., "flip" phenomena.	N =198	Ellume Ltd. COVID-19 Home Test 2/11/21 [12/15/20]	**95** [82–99, Δ = 17]	**97** [93–99,Δ = 6]	Lateral flow, fluorescence, instrument read, NCP Ag. OTC. Mid-turbinate nasal swab. Self-collected. ≥16 years, or adult-collected, ≥2 years. Home testing, screening. Smartphone assisted. *FP recall of* $>2(10)^6$ *products 2/24/21 through 8/11/21.*
	**Tier 2** (1)				
Highest PPA. Smallest sample size. NPA 100%, FP 0, and PPV 100%.	N = 128	SDBiosensor 1/5/22 [12/24/21] COVID-19 At-Home Test (Roche Diagnostics)	**95.3** [84.5–98.7, Δ = 14.2]	**100** [95.7–100, Δ = 4.3]	Lateral flow, visual read, serial screening. OTC. AN. Self-collected ≥14 years, adult-assisted ≥2 years, <6 days with symptoms. If without perform twice >24, <48 h between tests. NCP. "Negative results do not rule out SARS-CoV-2."
**Statistics**					
**Median, range [low-high]**	**N = 227.5** [128–597]		**86.6** [83.5–95.3, Δ_M = 11.8]	**99.25** [97–100, Δ_M = 3]	Median performance is sub-tier. Highest PPA and NPA achieve Tier 2.
**Mean [SD]**	**281.6** [157.9]		**88.43** [4.55]	**98.98** [1.02]	

**Part II. Molecular**	**Tier, Sample Size, and Cost**	**Company, Product, EUA LOA Date [Earliest Date]**	**Company PPA (%) Claim [CI, Δ]**	**Company NPA (%) Claim [CI, Δ]**	**Specimen Type, Age, and Time Interval/ Protocol for Specimen Collection [plus notes]**

Diagnostics, Details					
	**Tier 1** (2)				
RT-LAMP and lateral flow strip. ORF1ab region of the SARS-CoV-2 genome.	N = 112 Test & processing hub $75.	Detect COVID-19 Molecular Non-prescription Home Test 1/12/22 [10/28/21]	**90.9** [76.4–96.9, Δ = 20.5]	**97.5** [91.2–99.3, Δ = 8.1]	AN swab. Self-collected ≥14 or adult assisted ≥2 years. Without symptoms performed twice >24, <48 h between tests. Apparent FPs due to subject misinterpretation; app modified to reduce this error. Testing time 55–65 min.
RT-LAMP. Non-overlapping regions of the N gene.	N = 404 Overall N = 101 (1) N = 303 (2) Single-use test kit $75.	Lucira CHECK-IT COVID-19 Test Kit 4/9/21 [4/9/21]	**91.7** [85.6–95.8, Δ = 10.2] (1) 94.1 [85.5–98.4] (2) 90.1 [81.5–95.6]	**98.2** [95.8–99.4, Δ = 3.6] (1) 98.0 [89.4–99.9] (2) 98.2 [95.5–99.5]	AN swab. Symptomatic (1) and asymptomatic (2). PPAs exceed Tier 1 threshold of 90%. NPAs meet the Tier 2 threshold of 97.5%. Testing time 30 min.
	**Tier 2** (1)				
"Isothermal nucleic acid amplification test." Nucleocapsid N region of the SARS-CoV-2 virus.[8]	N = 271 Overall N = 138 (1) N = 133 (2) Test $61.75 to $65 each. Reader $249.	Cue Health COVID-19 Test for Home and OTC Use. 2/9/22 [3/5/21]	**97.4** [86.5–99.5, Δ = 13.0] **(**1) 96.4 [82.3–99.4] (2) 100 [72.2–100]	**99.1** [96.9–99.8, Δ = 2.9] (1) 98.2 [93.6–99.5] (2) 100 [97.0–100]	AN swab. Self-collected adult or assisted ≥2 years. Symptomatic (1) and asymptomatic (2). Testing time 20 min. A clinical evaluation of 292 outpatients in a community drive through showed sensitivity of 91.7% and specificity of 98.4% for S and A (with recent exposure) adult subjects.[8]
**Statistics**					
**Median, range [low-high]**	**N = 271** [112–404]		**91.7** [90.9–97.4, Δ_M = 6.5]	**98.2** [97.5–99.1, Δ_M = 1.6]	Median and mean performance are both Tier 1.
**Mean [SD]**	**262.3** [146.2]		**93.3** [3.54]	**98.3** [0.80]	

Abbreviations: Δ, magnitude in % of the 95% CI, i.e., high minus low limits; Δ_M, span of the median range; Ag, antigen; A, asymptomatic; AN, anterior nares; CI, 95% confidence interval with upper and lower bounds in percent; COVID-19, coronavirus disease 2019; EUA, Emergency Use Authorization; FDA, Food and Drug Administration (USA); RT-LAMP, reverse transcription loop-mediated isothermal amplification; LOA, letter of authorization; NA, not applicable; NCP, nucleocapsid protein; NPA, negative percent agreement; O, overall; OTC, over the counter; PPA, positive percent agreement; PPV, positive predictive value; and S, symptomatic. **Notes:** (a) Tier sensitivity/specificity (%) comprise: (1) 90/ 95;(2) 95/ 97.5; and (3) 100/ ≥99. (b) Data are reported as they appear in FDA EUA authorization letters and Information for Users under "In Virto Diagnostic EUAs—Antigen Diagnostic Tests for SARS-CoV-2, Home Testing posted before the end of 2021. (c) Prescription, telehealth, and home collection EUAs are not listed. (d) SD Biosensor "Standard Q COVID-19 Ag Home Testing" was recalled 31 January 2022.

Table S2. COVID-19 Antigen Test Performance for Symptomatic and Asymptomatic Subjects in Community Settings.

Tier (N), Author, Journal, Year, & Modality	Sensitivity (%) [95% CI] (Ranked)	Specificity (%) [95% CI]]	Antigen Assay	Sample Size (N), Sites, and Notes
**Part I. Point-of-Care Testing**				
**Sub-tier (29)**				

**Alghounaim**<br>*Frontiers Med*<br>2021	**30.6** [19.6–43.7] [O]<br>77.8 [40–97.2] [S]	**98.8** [97.8–99.4] [O]<br>94.7 [74–99.9] [S]	Standard Q RAgT, SD Biosensor. ONP swabs.	N = 972. Community screening 10 days, 28 symptomatic. “Most infections (77.4%) were asymptomatic.” Kuwait.
**Garcia-Finana**<br>*BMJ*<br>2021	**40** [28.5–52.4] [A]	**99.9** [99.8–99.99] [A]	Innova SARS-CoV-2 Ag rapid LFT.	N = 5869. Asymptomatic at 48 sites, 200 each in Liverpool, UK.
**Allan-Blitz**<br>*J Clin Micro*<br>2021	**47.5** [39.1–56.1] [O]<br>47.7 [35.2–60.5] [S]<br>54.4 [39.0–69.1] [A]	**100** [99.3–100] [O]<br>100 [98.7–100] [S]<br>99.8 [98.7–100] [A]	BinaxNOW COVID-19 rapid Ag Card, Abbott. AN swabs. Results for AN PCR.	N = 834 [O], 276 [S], & 422 [A]. Four publicly accessible testing sites across Florida.
**Mungomklang**<br>*Am J Trop Med Hyg*<br>2021	**47.97** [36.10–59.96] [A]	**99.71** [99.15–99.94] [A]	“Rapid SARS-CoV-2 antigen test.” NP swabs.	N = 1100. Asymptomatic migrant workers during case finding in Samut Sakhon, Thailand.
**Jakobsen**<br>*J Pathol Micro Immuno Scand*<br>2021	**48.5** [NR] [O]<br>56.2 [NR] Ct < 33<br>63.9 [NR] Ct < 30	**100** [NR] [O]<br>100 [NR] Ct < 33<br>100 [NR] Ct < 30	Standard Q COVID-19 Ag Test. AN swabs.	N = 7074. Low 0.9% prevalence public test center, Copenhagen, Denmark.
**Prince-Guerra**<br>*MMWR*<br>*2021*<br>**Almendares**<br>*J Clin Micro*<br>2021	**52.5** [46.7–58.3] [O]<br>64.2 [56.7–71.3] [S] 39.6 [26.4–54.0] [A]	**99.9** [99.7–100.0] [O]<br>100 [99.4–100.0] [S] 99.8 [99.5–100.0] [A]	BinaxNOW COVID-19 Ag Card. AN swabs.	N = 3419 with 827 symptomatic & 1968 asymptomatic. Two community-based testing sites in Arizona.
**Stohr**<br>*Clin Micro Infect*<br>2021	**55.6** [50–5–60.7] [O]<br>49.1 [41.7–56.5] (1)<br>61.5 [54.6–68.3] (2)	**99.8** [99.6–99.9] [O]<br>99.9 [99.7–100] (1)<br>99.7 [99.4–99.9] (2)	(1) BD Veritor System for Rapid Detection of SARS-CoV-2 (VRD). (2) Roche SARS-CoV-2 Ag detection test. Nasa; swabs.	N = 3201. N = 1595 (1) & 1606 (2). Testing kits obtained from a MHS community center, Netherlands, then self-testing performed at home.
**Frediani**<br>*Nature Sci Report*<br>2021	**57 [**37–76] (1 < 7 days) [O]<br>**74** [64–82] (2 < 7 days) [O]	**100** [79–100] (1 < 7 days) [O]<br>**99** [97–100] (2 < 7 days) [O]	BinaxNOW COVID-19 rapid Ag Test. AN swab.	Assessment of self-administration. N = 44 self- or parent-collected (1) & 297 staff-collected (2).
**Pollock NR**<br>*Open Forum Infect Dis*<br>2021	**57.7** [51.1–64.1] [O]<br>84.3 [71.4–93.0] [S < 7 days] &<br>50.0 [41.0–59.0] [A] adults.<br>85.7 [42.1–99.6] [S < 7 days] &<br>51.4 [34.4–68.1] [A] children.	**98.3** [97.5–99.0] [O]<br>97.5 [92.8–99.5] [S < 7 days] &<br>99.1 [98.3–99.6] [A] adults.<br>85.0 [62.1–96.8] [S < 7 days] &<br>97.8 [94.5–99.4] [A] children.	Access Bio CareStart COVID-19 Antigen Test	N = 1498, 1245 adults & 253 children. Sensitivity 79.6% for Ct≤30. FP were 21/234 tests. Community testing site in MA.
**Pilarowski**<br>*J Infect Dis* 2021	**57.7** [36.9–76.6] [O]<br>93.3 [68.1–99.8] when Ct ≤ 30.	**100** [99.6–100] [O]<br>99.9 [99.4–99.9] when Ct ≤ 30.	BinaxNOW COVID-19 rapid Ag Test.	N = 878. Public plaza in San Francisco, California, over 3 days,

			Nasal swab by technician.	symptomatic and asymptomatic (40%).
**Boum**<br>*Lancet*<br>2021	**58** [53–64] [O]<br>80.0 [71.0–88.0] [S first 7 days]<br>37.0 [27.0–48.0] [A]	**94** [88–97] [O]<br>NR	SD Biosensor, South Korea. NP swabs. (Data from table on page 1094.)	N = 1090 [O]. Community screening in Cameroon.
**Drevinek**<br>*Epid Mikrobiol Immuno*<br>2020	**66.4** [59.9–72.2] [O]<br>73.8 [66.7–79.9] [S]<br>43.6 [31.4–56.7] [A]	**100** [99.0–100] [O]<br>NR<br>NR	PanBio Ag Test, Abbott. NP swabs.	N = 591. Large-scale testing, single site, Prague, Czech Republic. Symptomatic (290) and asymptomatic (301) subjects.
**Pollreis**<br>*PLoS ONE*<br>2021	**67.6** [50.2–81.9] [O]<br>50.0 [6.8–93.2] [A subset]	**100.0** [97.9–100.0] [O]<br>100.0 [89.4–100.0] [A subset]	BinaxNOW COVID-19 Test Ag Card. Nasal swabs.	N = 214. Local public health district, rural population, Idaho (1). N = 14 asymptomatic subset (2). 82.7% symptomatic.
**Jakobsen**<br>*medRxiv*<br>2021	**69.7** [NR] [O]<br>78.8 [NR] [S]<br>49.2 [NR] [A]	**99.5** [NR] [O]<br>98.9 [NR] [S]<br>99.6 [NR] [A]	Standard Q COVID-19 Ag Test. OP swabs.	N = 4697. 705 self-reported S & 3008, A. Public test center, Copenhagen, Denmark.
**Nalumansi**<br>*In J Infect Dis* 2021	**70.0** [60–79] [O]<br>(More likely positive with qRT–PCR Ct 29, then sensitivity of 92%.)	**92** [87–96] [O]	Standard Q COVID-19 Ag Test. NP swabs.	N = 262. Males (89%) 14% of whom were symptomatic at treatment centers in Uganda.
**Fernandez-Montero**<br>*eClin Med*<br>2021	**71.43** [56.74–83.42] [A]	**99.68** [99.37–99.86] [A]	SARS-CoV-2 Rapid Antigen Test, Roche Diagnostics.	N = 2542. Asymptomatic adults, semi-closed community,<br>Univ. of Navarra, Spain.
**Gremmels**<br>*eClin Med*<br>2021	**72.6** [64.5–79.9] [Utrecht, S]<br>81.0 [69.0–89.8] [Aruba, S]	**100** [99.7–100] [Utrecht, S]<br>100 [99.7–100] [Aruba, S]	Panbio COVID-19 Ag Rapid Test, Abbott. NP swabs.	U: N = 1367 in Utrecht, Netherlands. N = 208 in Aruba. COVID-19. 16+ years at community testing centers.
**Jian**<br>*Int J Infect Dis*<br>2021	**76.39** [64.91–85.60] [O]	**99.26** [98.78–99.58] [O]	COVID-19 Antigen Rapid Test, Eternal Materials, Taipei, Taiwan. NP swabs.	N = 2096. High-throughput community testing site in Wanhua District of Taipei, Taiwan. Both S & A.
**Shah**	**77.2** [72.4–81.6] [O]<br>81.4 [76.8–85.5] (Or)<br>78.6 [73.4–83.3] [S]<br>68.8 [53.7–81.3] [A]	**99.6** [99.2–99.8] [O]<br>99.6 [99.2–99.8] (Or)<br>99.8 [99.2–100.0] [S]<br>99.4 [98.6–99.8] [A]	BinaxNOW COVID-19 rapid Ag Test. Nasal swab. 2nd swab at 30 min for repeat testing same day.	N = 2110. Overall & repeat (Or). 1188 S & 877 A in community testing site in Wisconsin.
**Pollock NR**<br>*J Clin Micro*<br>2021	**77.4** [72.2–82.1] [O]<br>96.5 [90.0–99.3] [S < 7 days] &<br>70.2 [56.6–81.6] [A] adults.	**99.4** [99.0–99.7] [O]<br>100 [98.6–100.0] [S< 7 days] &<br>99.6 [98.9–99.9] [A] adults.	BinaxNOW COVID-19 Ag Card. 20 tests per hour.	N = 2308 with 1380 adults (71% A) & 928 children (89% A). FP were 12/2308 tests.

	84.6 [65.1–95.6] [S < 7 days] & 65.4 [55.6–74.4] [A] children.	100 [94.5–100.0] [S < 7 days] & 99.0 [98.0–99.6] [A] children.		Community testing site in Massachusetts.
**Ford** *J Ped Infect Dis Soc* 2021	**80.8** [75.9–85.1] [O] (Adults) 73.0 [55.9–86.2] (C) 75.9 [56.5–89.7] [SC] 57.1 [18.4–90.1] [AC]	**99.9** [99.5–100] [O] (Adults) 100 [98.1–100] (C) 100 [NR][SC] NR	BinaxNOW COVID-19 rapid Ag Test. Nasal swab.	N = 1807 adults. N = 217 children (C). Self-collected at community public testing site in Wisconsin.
**Nsoga** *PLoS ONE* 2021	**81** [74.2–86.6] [O]	**99.1** [96.9–99.9] [O]	Panbio COVID-19 Ag Rapid Test, Abbott. OP swabs.	N = 402. Screening center in Geneva, Switzerland. "Most had symptoms."
**Siddiqui** *Microbiol Spectrum* 2021	**81 [**75–86] [O] 87 [80–91) [S] 71 [61–80] [A] 82 [66–91] [AE] 64 [51–76] [ANE]	**99.8** [100–100] (sic) [O] > 99 [NR] all groups	BinaxNOW COVID-19 rapid Ag Test. Nasal swab.	N = 6099. Overall, symptomatic, & asymptomatic. Self-referred walk-up testing site.
**Drain** *Am J Clin Pathol* 2021	**82.1** [64.4–92.1] [A]	**100** [98.1–100] [A]	LumiraDx SARS-CoV-2 Ag Test. AN swabs.	N = 222. Asymptoma- tic adults & children at five clinic- & community-based sites in the US.
**Shrestha** *Kathmandu Univ Med J* 2020	**85** [NA] [A, day 5]	**100** [NA] [A, day 5]	"An Ag test kit for COVID-19."	N = 113. High risk close contacts in quarantine in province 3, Kathmandu, Nepal.
**Chiu** *Microbiol Spectr* 2021	**85.3** [75.6–91.6] [S < 5 days] 82.7 [72.6–89.6] self–collected. 84.2 [69.6–92.6] [A]	**94.9** [91.6–96.9] [S < 5 days] 96.4 [93.4–98.0] self–collected. 99.9 [99.9–100] [A]	INDICAID COVID-19 rapid antigen test, PHASE Sci. Intl. AN swabs. 20 min test.	N = 349 symptomatic in California communities & 22,994 asymptomatic in Hong Kong outbreak screening centers.
**Stokes** *Eur J Clin Micro Infect Dis* 2021	**86.1** [81.3–90.0] [S < 7 days]	**99.9** [99.5–100] [S < 7 days]	Panbio COVID-19 Ag Rapid Test, Abbott. NP swabs.	N = 1641. Sympto- matic subjects < 7 days, community assessment centers.
**Agarwal** *J Infect Dev Ctries* 2021	**89.7** [72.6–97.8] [O]	**99.5** [NR] [O]	Standard Q COVID-19 Ag test. Nasal swabs.	N = 467. Fever clinic in north India. 2 AFP. Compared to TrueNat, POC chip-based real-time portable PCR.
Tier 1 (2)				
**Kerneis** *Eur J Clin Micro Infect Dis* 2021	**94** [86–98] [O] 95 [87–99] [S] 88 [64–99] [A]	**99** [98–99] [O] 98 [96–99] [S] 99 [98–100] [A]	Standard Q COVID-19 Ag test. NP swabs.	N = 1109 with 459 symptomatic & 650 asymptomatic. Paris community screening centers.
**Van der Moeren** *PLoS ONE* 2021	**94.1** [71.1–100] [S] 78.9 [70.6–85.7] in qRT–PCR positive prequalified subjects	**100** [98.9–100] [S]	BD Veritor System for Rapid Detection of SARS-CoV-2 (VRD). NP swabs.	N = 352. Municipal Health Service test centers in the Netherlands. Symptomatic.

**Tier 2 (1)**				
**Drain**<br>*Infect Dis Ther*<br>2021	**97.5** [87.1–99.6] [O] NP swab (Tier 2)<br>**97.6** [91.6–99.3] [O] N swab (Tier 2)	**97.7** [94.7–99.0] [O] NP swab (Tier 2)<br>**96.6** [92.7–98.4] [O] N swab (Tier 1)	LumiraDx SARS-CoV-2 antigen test.	N = 512. Children & adults (81% S) in ten clinic- & community-based settings in US & UK cities.
**Statistics**	**Median [N, Range]**	**Median [N, Range]**		
**Overall [O]**	**69.85 [24, 30.6–97.6]**	**99.5 [24, 92–100]**		
**Symptomatic [S]**	**81.0 [19, 47.7–96.5]**	**99.85 [16, 85–100]**		
**Asymptomatic [A]**	**55.75 [20, 37–88]**	**99.70 [16, 97.8–100]**		
**Part II. Antigen Automated Instrument Tests**				
**Alghounaim**<br>*Frontiers Med*<br>2021	**43.3** [30.6–56.8] [O]<br>88.9 [51.8–99.7] [S]	**99.9** [99.3–100] [O]<br>100 [80.5–100] [S]	SARS-CoV-2 Ag assay on LIAISON XL, Diasorin. ONP swabs.	N = 972. Community screening 10 days, 26 symptomatic. Kuwait.
**Drevinek**<br>*Epid Mikrobiol Immuno*<br>2021	**62.3** [55.8–68.4] [O]<br>68.5 [61.1–75.0] [S]	**99.5** [98.0–99.9] [O]<br>NR	Standard F Ag FIA, SD Biosensor. NP swabs.	N = 591. Large-scale testing, single site, Prague, Czech Republic. Symptomatic (290) and asymptomatic (301) subjects.
**Van der Moeren**<br>*J Clin Virol*<br>2021	**73** [61.3–82.7] [S]	**100** [97.9–100] [S]	SARS-CoV-2 Ag assay on LIAISON XL LAI, Diasorin. ONP swabs.	N = 248. Community-dwelling symptomatic subjects at Municipal Health Service COVID-19 test centers, Netherlands.
**Gili**<br>*Int J Infect Dis*<br>2021	**100** [96–100] [O] for screening @ 1.645 pg/mL optimal cutoff. 92.6 [85.4–97.0] in smaller cohort @ 1.24 pg/mL optimal cutoff.	**94.8** [93.6–95.8] [O] For screening @ 1.645 pg/mL optimal cutoff. 90.8 [84.5–95.2] in smaller cohort @ 1.24 pg/mL optimal cutoff.	Lumipulse SARS-CoV-2 antigen assay, benchtop Fujirebio, Tokyo. NP swabs.	N = 1738 swabs collected in schools, prisons, elderly care homes, & from hospital healthcare worker surveillance in Italy.
**Statistics**	**Median [N, Range]**	**Median [N, Range]**		
**Overall [O]**	**62.3 [3, 43.3–100]**	**99.5 [3, 94.8–99.9]**		
**Symptomatic [S]**	**73 [3, 68.5–88.9]**	**100 [3, 100]**		
**Asymptomatic [A]**	**No Data**	**No Data**		

Abbreviations: A, asymptomatic; AE, asymptomatic with exposure; Ag, antigen; AN, anterior nasal; ANE, asymptomatic with no exposure; CI, 95% confidence interval; CLEIA, chemiluminescence enzymatic immunoassay; Ct, cycle threshold; FIA, fluorescence immunoassay; FN, false negative; FP, false positive; ID, infectious disease; LFT, lateral flow test; MHS, Municipal Health Services; N, nasal; NA, not available; NP, nasopharyngeal; NR, not reported; O, overall; ONP, oronasopharyngeal; OP, oropharyngeal; PCR, polymerase chain reaction; PPV, positive predictive value; qRT-PCR, quantitative reverse transcription PCR; RAgT, rapid antigen test; S, symptomatic; SARS-CoV-2, severe acute respiratory syndrome coronavirus-2; UK, United Kingdom; and US, United States. **Note:** Tier sensitivity/specificity (%) comprise: (1) 90/ 95; (2) 95/ 97.5; and (3) 100/ ≥99.

Table S3. COVID-19 Antigen Test Performance in Emergency Medicine.

Category, Author, Journal, and Year	Sensitivity (%) [95% Confidence Interval]	Specificity (%) [95% Confidence Interval]	Antigen Assay/ Specimen/ Testing Details	Sample Size (N) and Descriptive Notes
**I. Emergency Medicine (EM)**				
**Bianco**<br>*J Clin Virol*<br>2021	**90.3** [86.3–93.4] [O]<br>89.3 [84.2–93.3] [S]<br>92.1 [85–96.5] [A]	**92.1** [89.7–94.1] [O]<br>88.2 [72.5–96.7] [S]<br>92.3 [89.9–94.4] [A]	LumiraDx Platform, UK. Instrumented test runs on a portable, wall outlet or battery-powered multi-assay desktop platform. Nasal swab.	N = 907. Adult and pediatric patients in EDs and occupational medicine during the 2nd peak of the Italian pandemic.
**Burdino**<br>*J Virol Meth*<br>2021	**89.6** [NR] from Table 1. In text: 90.1 [86.2–93.1]	**99.4** [NR] from Table 1. In text: 99.4 [98.6–99.8]	LumiraDx SARS-CoV-2 Ag Test. Nasal swab.	N = 1232. Patients referred to the ER in a tertiary care hospital in Turin, Italy.
**Caruana**<br>*Microorganisms*<br>2021	**41.2** b–d [O]<br>**48.3** a [O]<br>43.3 b–d [S]<br>52.2 a [S]<br>33 a–d [A]	**99.5** a **99.7** b [O]<br>**99.5** c **99.7** d [O]<br>99 a, 99.5 b–d [S]<br>100 a, b, d [A]<br>99.5 c [A]	4 RAgTs evaluated: (a) Exdia, (b) Standard Q RAgT, (c) Panbio, & (d) BD Veritor.	N = 532 with S (293) and A (239). RAgT performed by lab technicians in the ER. (No CIs reported for sensitivity & specificity.)
**Caruana**<br>*New Microbes New Infect*<br>2021	**28.6** [NR] [A]	**98.2** [NR] [A]	Standard Q COVID-19 Rapid Antigen Test, SD Biosensor/Roche. NP swabs.	N = 116. Asymptomatic ED patients screened on admission to Morges, Switzerland community hospital. 2 FP results. PPV 50%.
**Cento**<br>*Viruses*<br>2021	**85** [82–89]<br>91 [86–95] for samples with Ct≤29.	**97** [96–98]	LumiraDx SARS-CoV-2 test. NP swab.	N = 960. ER admissions to a tertiary COVID-19 hospital. 50 FN confirmed by RT-PCR.
**Cerutti**<br>*J Clin Virol*<br>2020	**70.6** [NR]	**100** [NR]	Standard Q COVID-19 Ag (R-Ag) test, Roche Diagnostics. NP "secretions."	N = 330. Symptomatic patients in ERs of 2 infectious disease reference centers, North Italy.
**Ciotti**<br>*J Med Virol*<br>2021	**30.77** [17.02–47.57]	**100** [71.51–100.00]	Ag Respi-Strip test.	N = 50. Mixed ED & infectious diseases ward patients in Rome, Italy.
**Holzner**<br>*J Med Virol*<br>2021	**68.87** [±1.86] [O]<br>69.46 [±2.30] [S]<br>62.0 [±0.32] [A]<br>All & Ct < 30, 80.48	**99.56** [±0.26] [O]<br>99.51 [±0.34] [S]<br>97.63 [± 1.03] [A]<br>All & Ct <30, 99.56	Standard Q, Roche Diagnostics	N = 2375 with S (1539) & A (836). For 423 S & Ct < 30, sensitivity 79.67 [±2.06] & specificity 99.51 [±0.35].
**Koelman**<br>*Eur J Clin Micro ID*<br>2021	**65.3** [57.1–72.8]	**100** [96.9–100]	Romed lateral flow immunochromatographic assay, Netherlands	N = 150. ER patients presenting to a teaching hospital in Rotterdam, Netherlands.
**Leixner**<br>*Intl J Infect Dis*<br>2021	**69.2** [58.8–78.3] [S]<br>Ct < 25, 100.0<br>Ct < 30, 91.8	**99.7** [98.1–100.0] [S]	AMP Rapid Test SARS-CoV-2, Austria. NP Swab.	N = 392. Symptomatic patients presenting to the Edin Vienna, Austria. Median Ct 27.6.

**Leli**<br>*Intl J Infect Dis*<br>2021	**68.7** [60–9–75.5] [O]<br>81 [70.3–88.6] [S]<br>48.1 [34.5–62] [A]	**95.2** [93.1–96.7] [O]<br>98.4 [93.9–99.7] [S]<br>93.8 [90.4–96.1] [A]	LumiraDx SARS-CoV-2 Ag Test. NP Swab. (Median Ct for Ag+ was 23.9, and Ag-, 35.6.)	N = 792. Patients admitted to ED in Alessandra, Italy. Prevalence 21% overall; S (207), 38.2%; & A (377), 14.3%.
**Linares**<br>*J Clin Virol*<br>2020	**73.3** [62.2–83.8] [O]<br>86.5 [75.5–97.5] < 7 days [S]<br>54.5 [A]	**100** [NR]<br>[NR for S & A]	Panbio COVID-19 Rapid Test Device, Abbottt. Median Ct 23.3. NP swab.	N = 255. Symptomatic (72.1%) & asymptomatic with close contact in the ED & primary care in Madrid.
**Loconsole**<br>*BioMed Res Intl*<br>2021	**94.9** [91.9–97.0] [O]<br>95.8 [92.7–97.7] [S]<br>91.8 [81.9–97.2] [A]	**97.4** [96.5–98.1] [O]<br>96.4 [93.7–98.0] [S]<br>97.8 [96.8–98.3] [A]	Lumipulse CLEIA Ag Test on G1200 automated analyzer, Fujirebio, Tokyo, Japan, in 50–60 min.	N = 911 ED patients in Bari, Italy. NP samples processed quantitatively at regional reference laboratory. Not POC testing.
**Masia**<br>*Open Forum Infect Dis*<br>2020	**69** [53.3–80.1]<br>For [S], then 95 when Ct ≤ 25; 85 when Ct ≤ 30, & 89 for triad.	**100** [97.2–100]	Panbio COVID-19 Ag Rapid Test Device	N = 223. ED patients in Alicante, Spain. NP swabs. Sample types studied. (Symptom triad is fever, cough, & malaise.)
**Merrick**<br>*Infect Prev Pract*<br>2021	**70.7** [65.8–75.2]	**99.1** [98.1–99.6]	Lateral-flow Ag detection using Innova and SureScreen tests. AN swab.	N = 1422. St. Thomas' ED in central London. PPV of 97.7% and NPV of 86.4% with prevalence of 34.7%. 3 FP and 95 FN results.
**Mockel**<br>*Biomarkers*<br>2021	**75.3** [65.8–83.4] [S]	**100** [98.4–100] [S]	AGTEST Roche SD/Biosensor. Deep ONP specimens.	N = 281. Symptomatic adults in four adult EDs in Berlin. FN results (22) occurred in adult ED patients.
**Oh**<br>*J Korean Med Sci*<br>2021	**17.5** [8.8–32]<br>Ct≤30, 26.9 [13.7–46.1]<br>Ct≤25, 41.1 [21.6–64.0]	**100** [95.3–100]	Standard Q COVID-19 Ag Test, SD Biosensor, Suwon, South Korea	N = 118. ED and admitted population. "Clinical applicability for diagnosis higher if applied higher performance tests."
**Orsi**<br>*J Virol Meth*<br>2021	**93.3** [83.8–98.2] [S]<br>**86.7** [75.4–94.1] [S] (FIA)	**100** [92.9–100] [S]<br>**100** [92.9–100] [S] (FIA)	FREND COVID-19 Ag Rapid Diagnostic Test & Standard F COVID-19 Ag FIA versus RT-qPCR.	N = 110. Symptomatic patients who accessed the ER. Comparison study performed in laboratory within 8 h from swab arrival.
**Osterman**<br>*Med Micro Immuno*<br>2021	**50.34** [45.71–54.96]<br>**45.41** [40.48–50.43] (FIA)	**97.67** [95.63–98.77]<br>**97.78** [95.68–98.87] (FIA)	SARS-CoV-2. Rapid Antigen Test, Roche Diagnostics, & Standard Q COVID-19 Ag Test FIA versus RT-PCR. N/P swab.	N = 445/386 & 381/360 (FIA). ERs, care units, or employee test centers in Germany. Sensitivity "…markedly lower than reported by manufacturers."
**Thell**<br>*PLoS ONE*<br>2021	**77.9** [70.0–84.6] [S]<br>81.6 [68.0–91.2] when within 7 days	**98.1** [94.6–99.6] [S]<br>95.9 [86.0–99.5] when within 7 days	SARS-CoV-2 Rapid Antigen Test, Roche Diagnostics. NP swabs.	N = 296. 5 EDs in Austria. Suspected, symptomatic patients. 1% FP & 10.1% FN.
**Turcato**<br>*J Infection*<br>2021	Preliminary<br>80.3 [74.9–85.4] [O]	Preliminary<br>99.1 [98.6–99.3] [O]	SD Biosensor SARS-CoV-2 Rapid Antigen Test. NP swab.	N = 3410. S (991) & A (2419) patients who required ED evaluation in Merano, Italy.

Preliminary Report	89.9 [85.4–94.4] [S]<br>50.0 [36.0–63.0] [A]	97.6 [96.5–98.5] [S]<br>99.6 [99.1–99.9] [A]		*Preliminary - excluded from statistical summary.*
**Turcato**<br>*Am J Emer Med*<br>2022<br>Final Report	**82.9** [81.0–84.8] [O]<br>89.8 [88.0–91.5] [S]<br>63.1 58.4–67.8] [A]	**99.1** [98.8–99.3] [O]<br>97.6 [97.1–98.1] [S]<br>99.6 [99.5–99.7] [A]	SD Biosensor SARS-CoV-2 Rapid Antigen Test. NP swab.	N = 3899. S (1191) & A (2708) patients who required ED evaluation in Merano, Italy. 3.3% FN.
**EM Statistics**	**Median [N, Range]**	**Median [N, Range]**		
**Overall**	**68.79 [20, 17.5–94.9]**	**99.5 [20, 92.1–100]**		
**Symptomatic**	**77.9 [15, 43.3–95.8]**	**99.5 [14, 88.2–100]**		
**Asymptomatic**	**48.1 [11, 28.6–92.1]**	**98.85 [10, 92.3–100]**		
**II. Pediatric EM (ER & ED)**				
**Carbonell-Sahuquillo**<br>*J Med Virol*<br>2021	**70.6** [52.2–84.9] [S] (results in text)	**100** [98.9–100] [S]	Panbio COVID-19 Ag Rapid Test Device.	N = 357. Symptomatic ED pediatric patients in Valencia, Spain. Prevalence 15%.
**Denina**<br>*Intl J Med Sci*<br>2021	**94.1** [71.3–99.8]	**91.9** [86.9–95.5]	LumiraDx Platform. Nasal swab.	N = 191. Pediatric patients in ED in Turin, Italy with or without symptoms/exposure.
**Gonzalez-Donapetry**<br>*Ped Infect Dis J*<br>2021	**77.78** [51.92–92.63] [S]	**100** [98.88–100] [S]	Panbio COVID-19 Ag Rapid Test Device. NP swabs.	N = 440. Hospital Univ. La Paz symptomatic pediatric ED patients who meet COVID-19 criteria.
**Jung**<br>*Frontiers Pediatrics*<br>2021	**87.9** [71.8–96.6] [S]<br>Ct > 25, 63.6 [30.8–89.1]	**98.5** [96.3–99.6] [S]<br>Ct > 25, 99.6 [98.0–100.0]	BIOSYNEX COVID-19 Ag BSS	N = 308. Symptomatic pediatric patients 0–17 years in ED and primary care in France. Prevalence 10.7%
**Lanari**<br>*Viruses*<br>2021	**53.8** (1) [35.4–71.4]<br>**86.4** (2) [75.0–93.9]	**99.7** (1) **[**98.4–100]<br>**98.3** (2) [97.1–99.1]	(1) COVID-19 Ag FIA kit. (2) AFIAS COVID-19 Ag kit. NP swab.	N = 1146. Pediatric patients admitted to the Emergency Unit of IRCCS-Polyclinic of Sant'Orsola, Bologna.
**Mockel**<br>*Biomarkers*<br>2021	**72.0** [53.3–86.7] [S]	**99.4** [97.3–99.9] [S]	AGTEST Roche/SD Biosensor. Deep ONP specimens.	N = 202. Pediatric patients in one ED in Berlin. FN (7) & FP (1) results occurred.
**Quentin**<br>*Clin Micro Biol Infect*<br>2021	**69.57** [54.25–82.26]<br>82.86 [66.35–93.44] [S]<br>27.27 [6.02–60.97] [A]	**99.89** [99.41–100]<br>99.77 [98.74–99.99] [S]<br>100 [99.27–100] [A]	COVID19Speed-Antigen Test, BioSpeedia, France	N = 1009. Children presenting to the pediatric ED of the Univ. Hosp., Saint-Etienne, France. S (493). A (516) children had the most FN results.
**Reichert**<br>*Am J Infect Cont*<br>2021	**42.9** [9.9–81.6] [A]<br>Asymptomatic adults compared: 38.9 [17.3–64.3]	**99.4** [98.6–99.8] [A]<br>Asymptomatic adults compared: 99.7 [98.4–100]	SD Biosensor SARS-CoV-2 Rapid Antigen Test. NP swab.	N = 710. Asymptomatic (x 48 h) pediatric patients in ED. No known contacts 14 days.
**Villaverde**<br>J Pediatrics<br>2021	**45.4** [34.1–57.2] [S]	**99.8** [99.4–99.9] [S]	Panbio COVID-19 Ag Rapid Test Device. NP swabs.	N = 1620. Pediatric patients with symptoms within 5

				days in EDs at 7 medical centers.

Statistics	Median [N, Range]	Median [N, Range]
**Pediatric EM**	**71.3 [10, 42.9–94.1]**	**99.55 [10, 91.9–100]**
**Symptomatic**	**74.89 [6, 45.4–87.9]**	**99.79 [6, 98.5–100]**
**Asymptomatic**	**35.09 [2, 27.27–42.9]**	**99.7 [2, 99.4–100]**
**Not Stated**	**77.99 [4, 53.8–94.1]**	**99 [4, 91.9–99.89]**

Abbreviations: A, asymptomatic; Ag, antigen; AN, anterior nasal; CI, 95% confidence interval; CLEIA, chemiluminescence enzymatic immunoassay; Ct, cycle threshold; ED, emergency department; EM, emergency medicine; ER, emergency room; FIA, fluorescence immunoassay; FN, false negative; FP, false positive; NP, nasopharyngeal; NPV, negative predictive value; NR, not reported; O, overall; ONP, oronasopharyngeal; POC, point of care; RT-PCR, reverse transcription-polymerase chain reaction; PPV, positive predictive value; S, symptomatic; and SARS-CoV-2, severe acute respiratory syndrome coronavirus-2.

Table S4. COVID-19 Saliva Testing Sensitivity and Specificity Performance in Strictly Asymptomatic Subjects (no mixed populations)—Clinical Evidence.

Author, Journal, & Year	Sensitivity (%) [95% CI] (ranked)	Specificity (%) [95% CI]]	Sample Size (N), Context, & Descriptive Notes	Authors' Conclusions
**Nacher**<br>*PLoS ONE*<br>2021	**16.8** [10.1–25.6]	**98.9** [97.7–99.6]	N = 90. Prospectively enrolled asymptomatic with a testing indication in the Amazonian Forest. NPS RT-PCR reference method.	Saliva "…sensitivity–using NP samples as gold standard—went from very poor among asymptomatic patients…"
**Nacher**<br>*Font Med*<br>2021	**24** [est. CI, 15–33]	**NR**	N = 39% of 776 = 303 asymptomatic. Reported 46 in Figure 2. CI estimated from Figure 2. Sites and mobile testing in French Guiana up to 240 km in the Amazon forest. NPS RT-PCR RM.	"The sensitivity (of) saliva samples for asymptomatic persons seemed insufficient… without any temporal indication about the onset of infection…"
**Marx**<br>*Clin Infect Dis*<br>2021	**28.5** [8.2–64.1]	**99** [97.0–99.7]	N = 299 asymptomatic. Testing at community testing events and homeless shelters, Denver. NPS RT-PCR RM.	"Among asymptomatic participants, sensitivity was low …"
**Bosworth**<br>*J Clin Virol*<br>2020	**33** [NR]	NR	N = 15 asymptomatic. Data also reported in Table 1 by Khiabani *Am J Infect Control* 2021. WHO E/RdRp gene RM and other assays.	"**…**saliva as an alternative sample type."
**Alkhateeb**<br>*Diag Micobiol Infect Dis*<br>2021	**36** [11–69]<br>22 [3–60] if low risk	**100** [3–100]<br>100 [29–100]	N = 12 asymptomatic & 12 at risk, i.e., afebrile & no comorbidities. RT-PCR NPS RM.	"…saliva's low sensitivity in asymptomatic SARS-CoV-2 infections …"
**LeGoff**<br>*Nature Sci Rep*<br>2021	**38** [23–55] RT–LAMP<br>**92** [78–98] RT–PCR	**97** [96–98] RT–LAMP<br>**97** [96–98] RT–PCR	N = 1027 asymptomatic (RT-LAMP) and 978 asymptomatic (RT-PCR),	"No difference in RT-LAMP sensitivity… between symptomatic

			compared to NPS RT-PCR with ≥1 gene targets.	and asymptomatic participants.”
**Igloi** *PLoS ONE* 2021	50.0 [18.7–81.3] (1) 60 [26.2–87.9] (2)	99.1 [96.8–99.9] (1) 99.6 [97.5–99.9] (2)	N = 5 for SD Biosensor SARS-Co-2 saliva RAgT vs. saliva RT-PCR RM (1). N = 6 for NPS RAgT versus saliva RT-PCR RM(2).	Asymptomatic subjects. Not included in statistical analysis, because of small sample sizes and use of RAgT for saliva.
**Lopes** *Viruses* 2021	**55** [NR] (On p. 7 in text.)	**98.3** [NR] (Calculated from data on p. 6 with corrected matches of 786 and 55% sensitivity.)	N = 821. Asymptomatic patients and healthcare workers with close contacts admitted to hematology ward. RT-PCR NPS RM.	“Symptomatic subjects showed significantly higher viral detection rate … compared with those asymptomatic...”
**Fernandez-Gonzalez** *J Clin Micro* 2021	**60** [27.4–86.3]	**NR**	N = 208. Self-collection of saliva by outpatient asymptomatic patients. RT-PCR NPS TM.	“…saliva is an acceptable specimen for the detection of SARS-CoV-2 in the community setting.”
**Nagura-Ikeda** *J Clin Micro* 2020	**63.35** [NR] (median of evaluation comparisons with 4 reference methods)	**NR**	N = 15 asymptomatic. Qiagen RT-qPCR, cobas SARS-CoV2, direct RT-qPCR, and RT-LAMP RMs LDT.	“…RT- qPCR, cobas SARS-CoV-2, RT-qPCR kits, and RT-LAMP showed sufficient clinical sensitivities for selective use in clinical settings and facilities.”
**Babady** *J Mol Diag* 2021	**63.6** [46.6–77.8] (oral rinse)	**96.9** [89.5–99.5] (oral rinse)	N = 285, 224 symptomatic, 35 asymptomatic, & 26 unknown. RT-PCR NPS RM.	“…no difference in detection rate across samples types between symptomatic and asymptomatic participants.”
**Chau** *Clin Infect Dis* 2020	**64** [NR]	NR	N = 11 asymptomatic. Data also reported in Table 1 by Khiabani *Am J Infect Control* 2021. NP-throat swabs with RT-PCR RM.	“Asymptomatic infection is common and can be detected by analysis of saliva.”
**Herrara** *Intl J Infect Dis* 2021	**78.1** [NR] 80.3 [73.6–86.0] if inconclusive results were eliminated.	**98.8** [NR] 99.5 [99.0–99.7] if inconclusive results were eliminated.	N = 2107. Asymptomatic healthcare and office workers in Mexico City. NPS Rt-PCR RM.	“…saliva is as effective as NP swabs for the identification of SARS-CoV-2-infected asymptomatic patients.”
**Yokota** *Clin Infect Dis* 2021	**87.5** [NR] (Calculated from Table 2 combined raw data.)	**99.8** [NR] (Calculated from Table 2 combined data.)	N = 1924. Asymptomatic contact tracing & airport. RT-PCR NPS RM.	“…saliva specimens had high sensitivity and specificity.”
**Kerneis** *Eur J Clin Micro Infect Dis* 2021	**88** [69–97] (1) **92** [74–99] (2)	**95** [94–97] (1) **97** [95–98] (2)	N = 812 asymptomatic using MGI-2 saliva procedure (1); & N = 848, Roche reference method (2). Asymptomatic data in Table 3.	“Diagnostic accuracy of…saliva NAAT is similar to NP NAAT, subject to compliance with protocols for saliva.”

**Vogels**<br>*Med (CelPress)*<br>2021	**89.5** [NR]	**99.9** [NR]	N = 3779. Asymptomatic & presymptomatic basketball NBA players & staff. Quest BioReference AN/OP swab RM.	Commercial evaluation of SalivaDirect in healthy individuals.
**Balaska**<br>*Diagnostics*<br>2021	**90** [94.6–99.6] (Calculated from collated screening data in Table 2.)	**100** [98.1–100] (Calculated from collated screening data in Table 2.)	N = 200. Screening of asymptomatic healthcare workers. Advanta Dx SARS-CoV-2 RT-PCR saliva-based assay evaluation study. NPS NeumoDx or Abbott RT-PCR RMs.	"Fluidigm Advanta Dx RT-PCR saliva-based assay may be a reliable diagnostic tool for… screening asymptomatic healthcare workers."
**Rao**<br>*Med Virol*<br>2021	**95** [83.8–100] (Estimated by the authors using a Bayesian latent class model.)	**99.9** [98.9–100] (Estimated by the authors using a Bayesian latent class model.)	N = 562 asymptomatic subjects in a detention center (210) and airport quarantine (352) in Kuala Lumpur, Malaysia. LCM: "Positive results of either test specimen (NP + OP swab) or random saliva was assumed as a perfect gold standard. "	"Self-collected saliva provides accurate surveillance testing of a community." "…estimated sensitivity and specificity of random saliva were higher than NP + OP swabs…."

**Statistics**	**Median [N, Range]**	**Median [N, Range]**		
	**63.6 [19, 16.8–95]**	**98.85 [14, 95–100]**		
	**Mean [SD], N**	**Mean [SD], N**		
	**62.9 [26.6], 19**	**98.4 [1.6], 14**		

Abbreviations: Ag, antigen; CI, 95% confidence interval; LAMP, loop-mediated isothermal amplification; LCM, (Bayesian) latent class model; LDT, lab developed test; NAAT, nucleic acid amplification test; NP, nasopharyngeal; NPS, nasopharyngeal swab; NR, not reported; OP, oropharyngeal; RT-PCR, reverse transcription polymerase chain reaction; RAgT, rapid antigen test; RM, reference method; SARS-CoV-2, severe acute respiratory syndrome coronavirus-2; and WHO, World Health Organization.

Table S5. Fundamental Definitions, Derived Equations, Ratios/Rates, Recursive Formulas, Predictive Value Geometric Mean-squared, and Prevalence Boundary.

**Eq. No.**	**Category and Equations**	**Dep. Var.**	**Indep. Var.**
	**Fundamental Definitions**		
1	x = Sens = TP/(TP + FN)	x	TP, FN
2	y = Spec = TN/(TN + FP)	y	TN, FP
3	s = PPV = TP/(TP + FP)	s	TP, FP
4	t = NPV = TN/(TN + FN)	t	TN, FN
5	p = Prev = (TP + FN)/N	p	TP, FN, N
6	N = TP + FP + TN + FN	N	TP, FP, TN, FN
	**Derived Equations**		
7	PPV = [Sens•Prev]/[Sens•Prev + (1-Spec)(1-Prev)], or<br>s = [xp]/[xp + (1-y)(1-p)]—symbolic version of the equation above	s	x, y, p
8	p = [s(y-1)]/[s(x + y - 1) - x]	p	x, y, s
9	x = [s(p-1)(y-1)]/[p(s-1)]	x	y, p, s
10	y = [sp(x-1) + s - px]/[s(1-p)]	y	x, p, s

11	NPV = [Spec•(1-Prev)]/[Prev•(1–Sens) + Spec•(1–Prev)], or t = [y(1–p)]/[p(1–x) + y(1–p)]	t	x, y, p
12	p = [y(1-t)]/[t(1 - x - y) + y]	p	x, y, t
13	x = [pt + y(1-p)(t-1)]/[pt]	x	y, p, t
14	y = [pt(x-1)]/[t(1-p)–1 +p]	y	x, p, t
	**Ratios**		
15	TP/FP = PPV/(1-PPV) = [Sens•Prev]/[(1-Spec)(1-Prev)], or [xp]/[(1-y)(1-p)]	TP/FP Ratio	x, y, p
16	FP/TP = (1-PPV)/PPV = [(1-y)(1-p)]/(xp)	FP/TP Ratio	x, y, p
17	FN/TN = (1-NPV)/NPV = [p(1-x)]/[y(1-p)]	FN/TN Ratio	x, y, p
	**Rates**		
18	R_{TP} = TP/(TP + FN) = x	R_{TP}	TP, FN
19	R_{FP} = FP/(TN + FP) = 1–Spec = 1- y	R_{FP}	TN, FP
20	R_{FO} = FN/(TN + FN) = 1–NPV = 1- t = [p(1-x)]/[p(1-x) + y(1-p)]	R_{FO}	x, y, p
21	R_{POS} = (TP + FP)/N	R_{POS}	TP, FP, N
	**Special Cases**		
	Recursive formulae for PPV (s_{i+1}) and NPV (t_{i+1})		
22a	s_{i+1} = [xp_i]/[xp_i + (1-y)(1-p_i)], where the index, i = 1, 2, 3…	s_{i+1}	x, y, p_i
22b	t_{i+1} = [y(1-p_i)]/[p_i(1-x) + y(1-p_i)]	t_{i+1}	x, y, p_i
	Prevalence when sensitivity is 100% (i.e., FN = 0)		
23	Prev = 1–[(1–N_+/N)/Spec], or p = 1- [(1-$POS_{\%}$)/y]	p	$POS_{\%}$, y
	PPV when sensitivity is 100%		
24	PPV = [Prev]/[Prev + (1-Spec)•(1-Prev)], or s = [p]/[p + (1-y)(1-p)]	s	y, p
	Predictive value geometric mean-squared (range 0 to 1)		
25	PV GM^2 = PPV•NPV = s•t = {[xp]/[xp + (1-y)(1-p)]}• {[y(1–p)]/[p(1–x) + y(1–p)]}	PV GM^2	x, y, p
	Prevalence boundary for a given R_{FO}		
26	PB = {y(1-t)/([(1-x) - (1-t)(1-x-y)]} = [yR_{FO}]/[(1-x) -R_{FO}(1-x-y)]	PB	x, t, R_{FO}
	Sensitivity when given specificity, R_{FO}, and PB		
27a	x = [PB-R_{FO}(y + PB-y•PB)]/[PB(1-R_{FO})]	x	y, R_{FO}, PB
	Sensitivity, given R_{FO} and PB, when specificity (y) is 100%		
27b	x = (PB-R_{FO})/[PB(1-R_{FO})]	x	R_{FO}, PB
	Accuracy (not recommended–see note)		
…	A = (TP + TN)/N = Sens•Prev(dz) + Spec•Prev(no dz)	A	TP, TN, N

Abbreviations: Dep. Var., dependent variable; Indep. Var., independent variable(s). Eq., equation. i, an index from 1 to 3 or more—the number of testing events. N, total number of people tested. N_+, number of positives (TP + FP) in the tested population. N-, number of negative (TN + FN) in the tested population. PB, prevalence boundary. $POS_{\%}$, (N_+/N), percent positive of the total number tested (same as R_{POS}). $NEG_{\%}$, (N−/N), percent negative of total number tested. Prev, prevalence (p); Prev(dz), same as p; Prev(no dz), prevalence of no disease. PPV, positive predictive value (s); NPV, negative predictive value (t). PV GM^2, square of the geometric mean of positive and negative predictive values, (PPV•NPV), expressed as a fraction from 0 to 1. p_{i+1}, p_i, indexed partition prevalence in the recursive formula for PPV and NPV. R_{FO}, the rate of false omissions. R_{FP}, false positive rate, aka false positive alarm—probability that a false alarm will be raised or that a false result will be reported when the true value is negative. R_{POS}, positivity rate. R_{TP}, true positive rate, the same as sensitivity. Sens, sensitivity (x); Spec, specificity (y). TP, true positive; FP, false positive; TN, true negative; FN, false negative. **Notes:** Sens, Spec, PPV, NPV, and Prev are expressed as percentages from 1 to 100%, or as decimal fractions from 0 to 1 by dividing by 100%. PV GM^2 was created for visual logistics comparisons of performance curves of diagnostic tests, not for point comparisons. If the denominators of derived equations become indeterminate, then revert to the fundamental definitions, Equations 1–6. The use of the formula for accuracy is not recommended, because of duplicity of values with complementary changes in sensitivity and specificity.

Table S6. Antigen Tests with FDA Emergency Use Authorization Ranked by Positive Percent Agreement.

Notes	Tier, Sample Size	Company, EUA Latest Date [Earliest Date], Product Name	PPA (%) [CI]	NPA (%) [CI]	Assay Method, Specimen Type, Collection, Interval for Procuring Specimen, Protocol [plus notes]
	**Sub-Tier**				
	Sub-tier N = 105	Ortho Clinical Diagnostics 3/16/21 [1/11/21] VITROS Reagent Pack	**80.0** [56.6–88.5] *[Lowest PPA]*	**100.0** [95.2–100.0]	Chemiluminescence immunoassay, instrument read; nasopharyngeal swab; healthcare provider; 7 days. [Second FDA EUA study available with PPA 86.2%, NPA 97.7%.]
	Sub-tier N = 226	Becton Dickinson 4/14/21 [7/2/20] Veritor System	**83.9** [67–93]	**100** [98–100]	Chromatographic digital immunoassay, instrument read, serial screening; nasal swab; healthcare provider; 5 days.
	Sub-tier N = 460	Abbott 4/20/21 [8/26/20] BinaxNow COVID-19 Ag Card	**84.6** [76.8–90.6] (PPA of 97.1% in N = 53 small set initially claimed.)	**98.5** [96.6–99.5]	Lateral flow, visual read; anterior nasal swab; healthcare provider; 7 days. Test once with guidance using NAVICA app for return flights [United Airlines]. Same N, PPA, & NPA as self test. Note temperature limits of 2–30 °C (35.6–86 °F) when travelling.
	Sub-tier N = 502	InBios 5/6/21 SCoV-2 Ag Detect Rapid Test	**86.67** [73.8—93.74]	**100** [98.53–100.00]	Lateral flow, visual read, serial screening; anterior nasal swab; healthcare provider; 5 days if symptoms. If none, test twice, 2–3 days apart, 24–48 h.
	Sub-tier N = 89	Qorvo Biotechnologies 4/13/21 Omnia SARS-CoV-2 Antigen	**89.47** [78.88–95.09]	**100** [89.28–100]	Bulk acoustic wave biosensor, instrument read, cartridges; anterior nasal swab; healthcare provider; 6 days.
	**Tier 1**				
	Tier 1	Celltrion 5/11/21 [4/16/21] DiaTrust COVID-19 Ag Rapid Test	**93.33** [78.7–98.2]	**99.03** [94.7–99.8]	Lateral flow, visual read, serial screening; nasopharyngeal swab; healthcare provider; 7 days if symptoms. If none, test twice, 2–3 days, 24–49 h.
	Sub-tier N = 180	Access Bio 4/15/21 [10/8/20] CareStart COVID-19 ANTIGEN	**93.75** [79.85–98.27]	**99.32** [96.27–99.88]	Lateral flow, visual read; nasopharyngeal swab; healthcare provider; 5 days. [PPA 87.18% performance inferior (NPA 100.00%) if anterior nasal swab. See FDA EUA.]
Multiplex	Tier 1 N = 125	Princeton BioMediTech 2/4/21 Status COVID-19/Flu	**93.9** [83.5–97.9]	**100** [95.2–100.0]	Lateral flow, visual read, multi-analyte; nasopharyngeal swab; healthcare provider; 5 days. [For Influenza A/B metrics, see FDA EUA.]
	Tier 1 N = 105	Celltrion USA 10/23/2020 Sampinute COVID Ag MIA	**94.4** [80.0–99.0]	**100.0** [88.0–100.0]	Magnetic force-assisted electrochemical sandwich immunoassay; nasopharyngeal swab; healthcare provider; 5 days.

	**Tier 2**				
Multiplex	Tier 2<br>N = 164	Quidel<br>10/2/20<br>Sofia 2 Flu + SARS Ag FIA	**95.2**<br>SARS –Cov–2<br>[84.2–98.7]	**100.0** SARS–Cov–2<br>[96.9–100.0]	Lateral flow, fluorescence, multi-analyte, instrument read; nasopharyngeal and nasal swabs; healthcare provider; 5 days. [For Influenza A/B metrics, please see FDA EUAs.]
Not plotted—see the other two Quidel tests.	Tier 2<br>N = 209	Quidel<br>4/1/21 [5/8/20]<br>Sofia SARS Ag IFA	**96.7**<br>[83.3–99.4]	**100.0**<br>[97.9–100.0]	Lateral low, fluorescence, instrument read on Sofia of Sofia 2 only; nasopharyngeal and nasal swabs; healthcare provider; 5 days if symptoms. If none, test twice, 2–3 days apart, 24–48 h.
	Tier 2<br>N = 138	Quidel<br>12/22/20 [12/18/20]<br>Quickview SARS Ag Test	**96.8**<br>[83.8–99.4]	**99.1**<br>94.9–99.8]	Lateral flow, visual read; anterior nares swab specimen; healthcare provider; 5 days. [Data for fresh specimens.]
	Tier 2<br>N = 166	Luminostics<br>12/7/20<br>Clip COVID Rapid Ag Test	**96.9**<br>[83.8–99.9]	**100**<br>[97.3–100]	Lateral flow immunoluminescent assay, instrument read; healthcare provider; anterior nasal swab; 5 days
	Tier 2<br>N = 141	DiaSorin<br>3/26/21<br>LIAISON SARS-CoV-2 Ag	**97.0**<br>[84.7–99.5]	**100**<br>[96.6–100]	Chemiluminescence immunoassay, LIAISON XL analyzer read; nasopharyngeal and direct nasal swab; healthcare provider; 10 days. [Wilson CI]
	Tier 1*<br>N = 25	LumiraDx UK<br>4/15/21 [8/18/20]<br>SARS-CoV-2 Ag Test	**97.6**<br>[91.6–99.3]	**96.6***<br>[92.7–98.4]	Microfluidic immunofluorescence assay, instrument read; nasal swab; healthcare provider; 12 days. [95% Wilson CI]
	Tier 2<br>N = 26	Quanterix<br>1/5/21<br>Simoa SARS-CoV-2 N Protein Ag Test	**97.7**<br>[92.03–99.72]<br>*[Best PPA]*	**100**<br>[90.75–100.0]	Paramagnetic microbead-based immunoassay; nasopharyngeal swab; healthcare provider; 14 days.

Abbreviations: Ag, antigen; CI, 95% confidence interval with upper and lower bounds in percent; CLIA, Clinical Laboratory Improvement Act; EUA, Emergency Use Authorization; FDA, Food and Drug Administration; FIA/IFA, immunofluorescence assay; MIA, magnetic force-assisted electrochemical sandwich immunoassay; NPA, negative percent agreement; PPA, positive percent agreement; SARS-CoV-2, severe acute re3spiratory syndrome-Coronavirus-2; and UK, United Kingdom. **Notes:** (a) Tier sensitivity/specificity (%) comprise: (1) 90/95; (2) 95/97.5; and (3) 100/≥99. (b) *One exception in the ranking by PPA—the NPA is not Tier 2. (c) Boldface PPA and NPA are used for computations underlying performance plots. (d) Data are reported exactly as they appear in FDA EUA Instructions for Users.

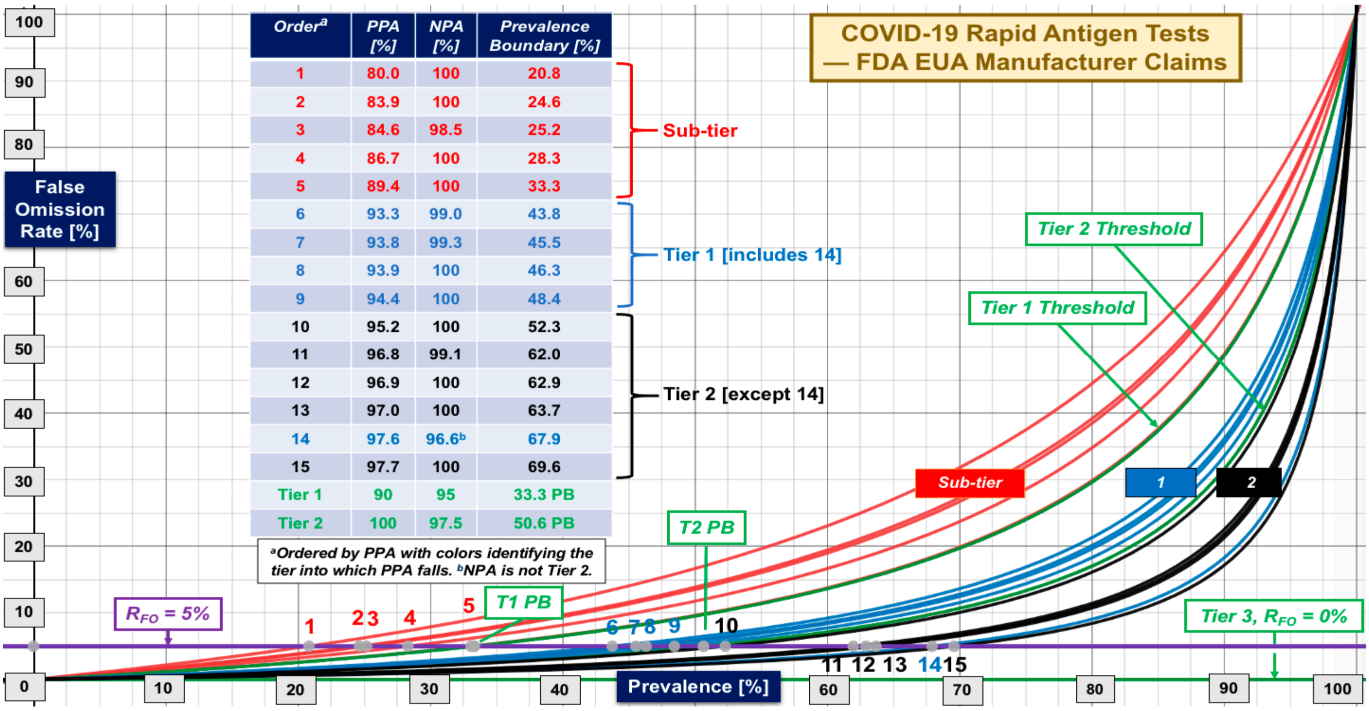

Figure S1. Rate of False Omissions for FDA EUA COVID-19 Rapid Antigen Tests. The purpose of this figure is to illustrate that manufacturer claims can attain Tier 2 performance. The figure integrates R_{FO} curves for the first 15 RAgTs that received FDA EUAs (see Table S6) listed in in order of ascending PPA. If prevalence exceeds the labeled boundaries, then 1 in 20 patients risk unknowingly spreading contagion. The three tiers were designed to allow progressive bracketing of false omission rates. Six of the 15 claims have prevalence boundaries above Tier 2 at 50.6%. These claims strengthen the case for using Tier 2 as an attainable and desirable performance standard for COVID-19 RAgTs. Abbreviations: NPA, negative percent agreement; PB, prevalence boundary; PPA, positive percent agreement; RAgT, rapid antigen test; and R_{FO}, false omission rate.
